# Sorption thermodynamic and kinetic study of Cu(II) onto modified plant stem bark

**DOI:** 10.1007/s11356-024-35194-6

**Published:** 2024-10-22

**Authors:** Yannice Tatiane da Costa Santos, Stefano Salvestrini, Clara Beatryz Gomes Vieira, Jorge Marcell Coelho Menezes, Antonio Junior Alves Ribeiro, João Victor Serra Nunes, Henrique Douglas Melo Coutinho, Diniz Maciel Sena Júnior, Francisco José de Paula Filho, Raimundo Nonato Pereira Teixeira

**Affiliations:** 1 Federal Institute of Education, Science and Technology of Ceará – campus Juazeiro do Norte, Av. Plácido Aderaldo Castelo, 1646, Juazeiro do Norte, Ceará 63040-540 Brazil; 2 Department of Biological Chemistry, Regional University of Cariri, R. Cel. Antonio Luis 1161, Crato, Ceará 63105000 Brazil; 3 Department of Environmental, Biological and Pharmaceutical Sciences and Technologies, University of Campania “Luigi Vanvitelli”, Via Vivaldi 43, 81100 Caserta, Italy; 4 Science and Technology Center, Federal University of Cariri, Av. Ten. Raimundo Rocha, 1639, Juazeiro do Norte, Ceará 63048-080 Brazil; 5 Analitycal Center, Federal University of Ceará – Campus Pici, Av. Humberto Monte, N/N, Fortaleza, Ceará 60440-900 Brazil

**Keywords:** *Ziziphus joazeiro*, Copper sorption, Sorption thermodynamics, Lignocellulose, Sorption isotherms, Sorption kinetics

## Abstract

In this study, four types of “Juá” stem barks (*Ziziphus joazeiro*) were investigated for the removal of Cu(II) from aqueous solutions. The tested samples included natural coarse barks, and barks washed with water, ethanol–water, and NaOH solutions. The solvent-modified materials simulated the waste of the industrial extraction of saponins from bark. The valorization of these processing residues as sorbents was evaluated. The NaOH-washed sorbent exhibited the highest sorption capacity for Cu(II) (maximum sorption capacity $\approx$ 32 mg g^−1^). Ion exchange process between copper and exchangeable surface cations and electrostatic attraction of copper with carboxylate and phenolate groups were identified as the primary sorption mechanisms. Desorption tests revealed that a large portion of the metal sorbed (80%) was easily released from the sorbent thus suggesting, in line with the proposed mechanisms, the existence of weak sorbate-sorbent interactions. The sorptive process was found to be exothermic (∆H° =  − 48.1 ± 13.5 kJ.mol^−1^) and thermodynamically favorable at lower temperatures.

**Graphical Abstract:**

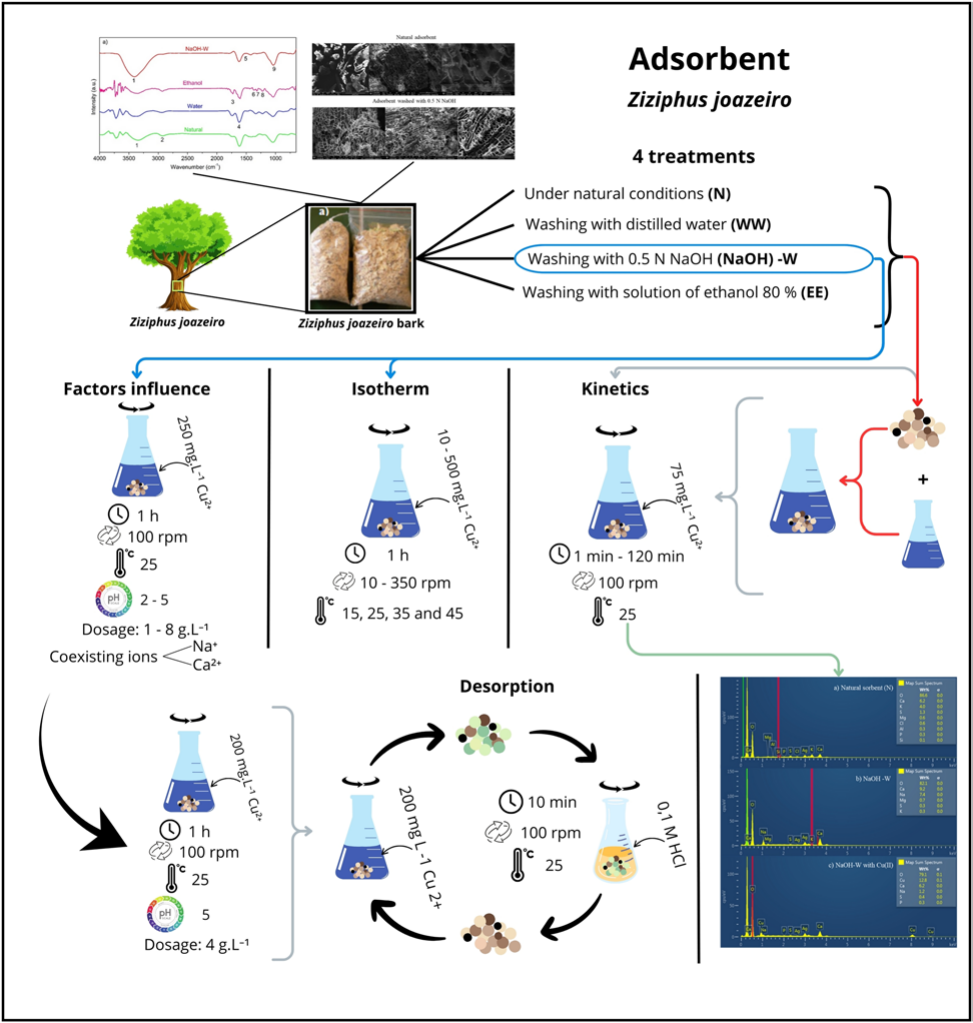

**Supplementary Information:**

The online version contains supplementary material available at 10.1007/s11356-024-35194-6.

## Introduction

Copper is an essential trace element in the human body and serves as a vital component of various enzymes. However, exceeding a certain threshold of copper ions in the body can result in damage to the liver, kidneys, capillaries, and central nervous system (Guo et al. 2020). Additionally, copper functions as a micronutrient or trace element for plants, playing a crucial role in growth, stress resistance, biosynthesis, and the functioning of diverse biomolecules such as carbohydrates, chlorophyll, nucleic acids, growth regulators, and secondary metabolites (Appenroth 2010). Both deficiencies and excesses of essential heavy metals can lead to diseases or abnormal conditions (Ali Redha 2020).

The main sources of water contamination by copper include industrial activities such as electroplating, metal cleaning and plating baths, fertilizers production, pulp and paper, board mills, wood pulp production, but also paints and pigments, petroleum industries, municipal and storm water runoff (Al-Rub et al. 2006; Zuo et al. 2018). For supply water, the main source of contamination comes from the corrosion of pipes (Cuppett et al. 2006).

Among the treatment techniques commonly employed to remove copper and other metals from aqueous environments (Akpomie and Conradie 2020; Bayuo 2021), several options exist, including physical methods such as ion exchange, membrane filtration, and ultrafiltration processes (Akpomie and Conradie 2020; Bayuo 2021). Chemical approaches involve techniques such as chemical coagulation, electrochemical methods, chemical precipitation, oxidation, and photochemical processes. Additionally, biological mechanisms, such as the bioaccumulation of metals in plants and microorganisms, are also utilized.

Apart from the above techniques, worth mentioning is also sorption mostly because of its ease of operation and high pollutants removal efficiency (An et al. 2015; Ge and Li 2018; Belaissa et al. 2022).

Over the years, many different materials have been proposed as sorbents and among them, lignocellulosic biomass (consisting of lignin, cellulose, and hemicellulose) has recently gained attention not only for its good sorption performance towards inorganic and organic pollutants but also because of its more environmental sustainability than that of synthetic sorbents. Another advantage of lignocellulose is that its chemical modification is easily achievable and cheap (Maia et al. 2021; Dias et al. 2023).

Among the variety of lignocellulosic sorbents, we can mention: “pequi” bark (Menezes et al. 2020; Coelho Menezes et al. 2021), leaves Neem (Zafar et al. 2013) *Azadirachta indica* A seeds (Costa et al. 2020), sugar cane bagasse (Ezeonuegbu et al. 2021), cassava root (Tho et al. 2021), *Moringa oleifera* seeds and leaves (Abatal et al. 2021), *Mangifera indica* waste biomass (Nadeem et al. 2014), barley straw (Saravanan et al. 2021), rice husk (Zhan et al. 2020), palm fiber (*Opuntia fuliginosa* and *Agave angustifólia*) (Flores-Trujillo et al. 2021), banana peel (Hu et al. 2021), avocado seeds (Dhaouadi et al. 2021a), corn cob (Tejada-Tovar et al. 2021), and *Trapa bispinosa's* peel (Zafar et al. 2019).

*Ziziphus joazeiro* is a biomass that has received relatively limited research attention. This popular tree, known as “Juá” is native to northeastern Brazil and has a rich history in traditional medicine, particularly for the treatment of gingivitis, bacterial infections, and respiratory diseases, with the roots, leaves, and bark commonly utilized for these purposes (Brito et al. 2015). Furthermore, the plant finds application in hygiene care, such as in the production of hair tonics and toothpaste, owing to the significant presence of the surfactant triterpene saponins (jujubosides) in its bark, ranging from 2 to 10% w/w (Carvalho 2007; Ribeiro et al. 2014; Rego 2019; do Nascimento et al. 2020).

Saponins and other bioactive compounds can be recovered from biomass by conventional methods using alkaline solutions (e.g., NaOH aqueous solutions) to breakdown/remove lignin, and alcohol/water extractant mixtures (e.g., ethanol–water) (Majinda 2012). These procedures generate undesired residues whose reutilization would lead to the minimization of waste with undeniable beneficial impact on the environment in line with the circular economy principles.

In this regard in the present work, the sorptive potential of “Juá” stem bark before and after chemical modifications simulating saponins extraction was explored for the removal of Cu(II) ions from water. The sorbent material was characterized determining the point of zero charge (pHpzc), via scanning electron microscopy with integrated energy-dispersive X-ray analysis (SEM–EDS) and infrared measurements (FTIR). Moreover, the kinetic and thermodynamic aspects of the sorption process were analyzed and elucidated.

## Materials and methods

### Preparation and characterization of the sorbents

The raw material, commonly known as “raspa de Juá” (Juá rasp), was obtained in its commercial form from the Public Market of Juazeiro do Norte, Ceará, Brazil. Chemical modifications were induced by washing the raw material (N) with distilled water (WW), an 80%/20% ethanol/water solution (EE), and a 0.5 N NaOH solution (NaOH-W), as shown in Table 1. The sorbents obtained according to the procedure described above simulate the solid waste generated for the industrial extraction of saponins from biomass. In the perspective of real water treatment applications, a relatively large size was selected for the sorbent particles (> 300 μm) in order to limit liquid flow resistance in column sorption processes and to easily recover the sorbent after batch tests.

**Table 1:** Chemical modification processes of the stem bark

Sorbent	Particle size (µm)	Preparation procedure
Natural (N)	1180 to 300 d_90_: 950 μm	Raw bark without changes
Washed with water (WW)		55 g of bark were washed manually with 2 L of distilled water and dried in oven at 103 °C for 24 h
Washed with ethanol (at 80%) (EE)		10 g of bark were submerged in 100 mL of Ethanol at 80% for 72 h, followed by washing with distilled water and dried in oven at 103 °C for 24 h
Washed with 0.5 N NaOH (NaOH-W)		5 g of bark were washed with 125 mL of 0.5 N NaOH solution for 1 h, under 200 rpm stirring, followed by rinsing in 120 mL of distilled water under 200 rpm stirring for 1 h and dried in oven for 1 h at 50 °C

The protocol for the determination of the pH at zero charge (pH_pzc_) was adapted from the methodology described by Regalbuto and Robles (Regalbuto and Robles 2004). In this method, 0.1 g of each sorbent was placed in contact with 20 mL of solution at different pH values ranging from 1 to 13. The mixtures were stirred at 100 rpm using a Nova Ética model 109–1 shaker for 2 h. The pH_pzc_ was calculated by plotting a graph of the pH difference (ΔpH = pH_initial_ – pH_final_) versus pH_initial_, and the pH_pzc_ was determined as the intersection point of the resulting curve with the x-axis (Hafshejani et al. 2015).

The samples, both before and after the sorption process, were examined using scanning electron microscopy (SEM) integrated with energy-dispersive X-ray spectroscopy (EDS) on a Quanta 450-FEG – FEI instrument. To analyze the functional groups and structure of the sorbents, infrared spectra were recorded using a FTIR spectrometer (FTIR Cary 660 Agilent) with an ATR (Ge) setup, 16 accumulations and a resolution of 4 cm^−1^.

The saponins content was determined by adapting the Afrosimetric Index for visual identification of foam height after agitation of diluted samples in test tubes. To this end, 0.5 g of sample was heated in 100 mL of water between 80 °C and 90 °C. The extract was diluted in test tubes with distilled water (10—100%) and manually agitated for 15 s; the saponins content was determined by measuring the persistence of foam with a minimum height of 1 cm (Araújo and Salles 2020).

### Sorption kinetic experiments and modelling

The kinetic tests were carried out in batch mode at 25 °C in 125-mL Erlenmeyer flasks by mixing 0.1 g of sorbent with 25 mL of a Cu(SO_4_)0.5H_2_O solution, Cu(II) concentration = 75 mg L^−1^, buffered at pH 5.2 with acetate buffer. The batches were stirred at 100 rpm on a Nova Ética model 109–1 shaker and, at predetermined times, small aliquots of supernatant were collected, filtered through fast-speed filter paper, and analyzed for the concentration determination of residual Cu(II) in the liquid phase by Flame Atomic Absorption Spectroscopy (FAAS) using a Varian SpectrAA 50B spectrometer.

The sorptive capacity (*q*) of the sorbents and the percentage of Cu(II) removal (*E(%)*) were determined using Eqs. (1) and (2), respectively:

1 $$q ({mg g}^{-1})=\frac{({C}_{0}- {C}_{t})V}{W}$$

2 $$E(\%)=\frac{({C}_{0}- {C}_{t})}{{C}_{0}}\times 100$$

where *C*_*0*_ and *C*_*t*_ are the initial and the concentration of Cu(II) at time *t*, whereas *W* and *V* are the sorbent mass (g) and liquid volume (L), respectively.

In order to investigate the rate and mechanism of the metal uptake, pseudo-first order (PFO), pseudo-second order (PSO), Elovich and Boyd kinetic models (Table 2) were used.

**Table 2:** Nonlinear equations and kinetic parameters applied in this study

Kinetic Models	Nonlinear equations	Parameters
Pseudo-first order equation (PFO) (Lagergren 1898; Ho 2004)	${q}_{t}={q}_{e}\times (1-{e}^{-{k}_{1}\times t})$	*q*_*t*_: Sorption capacity at any time (mg g^−1^) *q*_*e*_: Sorption capacity at equilibrium (mg g^−1^) *k*_*1*_: Pseudo-first order sorption rate constant (min^−1^) *t*: Time (min)
Pseudo-second order equation (PSO) (Ho and McKay 1999)	${q}_{t}= \frac{{{q}_{e}}^{2}\times {k}_{2} \times t}{1+{q}_{e} \times {k}_{2}\times t}$	*k*_*2*_: Pseudo-second order sorption rate constant (g mg^−1^ min^−1^)
Elovich’s kinetic model (Elovich and Zhabrova 1939; Taylor and Thon 1952; Low 1960; Tseng et al. 2022)	$q_t=\frac1\beta ln\;(1+\alpha\beta t)$	*α*: Initial sorption rate (mg g^−1^ min^−1^) *β*: Desorption constant (mg g^−1^)
Boyd (Diffusion intraporo model) (Boyd et al. 1947; Reichenberg 1953)	$$\begin{array}{l}\begin{array}{ll}If\;F<0.85:&Bt=\left(\sqrt\pi-\sqrt{(\pi-(\frac{\pi^2F}3})\right)^2\\If\;F>0.85:&Bt=-0.49770-\mathrm{In}\;(1-F)\\F=\frac{q_t}{q_e}&B=\frac{\pi^2D}{r^2}\end{array}\end{array}$$	*D*: Coefficient for effective diffusion (cm^2^ min^−1^) *r*: Radius particle (cm) *B*: Boyd model constant (min^-1^)

### Sorption equilibrium experiments and modeling

The equilibrium experiments were performed in duplicate, using 10 mL of Cu(SO_4_)0.5H_2_O (Cu(II) concentration ranging from 10 to 462 mg L^−1^) buffered with acetate buffer (final pH 5.2). The solution was put in contact with 0.05 g of the sorbent for 60 min (time sufficient to attain a stable Cu(II) concentration for all the experimental conditions tested) under stirring at 100 rpm and at different temperatures (15 °C, 25 °C, 35 °C, and 45 °C). Afterwards, the samples were analyzed as already described for the kinetic experiments (see Sect. 2.2). The nonlinear Langmuir, Freundlich, Temkin II, and Sips models (see Table 3) were used to fit the sorption experimental data at equilibrium.

**Table 3:** Equilibrium isotherm models applied to experimental data for modeling Cu(II) sorption

Isotherm Models	Nonlinear equations	Parameters
Langmuir (Langmuir 1916)	${q}_{e}=\frac{{q}_{max}\times {K}_{L}\times {C}_{e}}{1+{K}_{L}\times {C}_{e}}$	*q*_*e*_: Sorption capacity at equilibrium (mg g^−1^) *q*_*max*_: Maximum biosorption capacity teorical for monolayer (mg g^−1^) *K*_*L*_: Langmuir constant (L mg^−1^) *C*_*e*_: Ion final concentration in solution (mg L^−1^)
Freundlich (Freundlich 1906)	${q}_{e}={K}_{F}\times {{C}_{e}}^\frac{1}{n}$	*q*_*e*_: Sorption capacity at equilibrium (mg g^−1^) *C*_*e*_: Ion final concentration in solution (mg L^−1^) 1/*n*: Heterogeneity factor *K*_*F*_: Freundlich constant (mg g^−1^) (L mg^−1^)^1/n^
Temkin II (Chu 2021)	${q}_{e}={q}_{T}\times ln(1+{K}_{T}\times {C}_{e})$	*q*_*e*_: Sorption capacity (mg g^−1^) *K*_*T*_: Temkin constant (L mg^−1^) *C*_*e*_: Ion final concentration in solution (mg L^−1^) *q*_*T*_: surface capacity for contaminant sorption per unit binding energy (mg g^−1^)
Sips (de Vargas et al. 2023)	${q}_{e}=\frac{qmax\times {Ks \times {C}_{e}}^{\beta s}}{1+{Ks \times {C}_{e}}^{\beta s}}$	*q*_*e*_: Sorption capacity at equilibrium (mg g^−1^) *q*_*max*_: Maximum sorption capacity (mg g^−1^) *Ks*: Sips equilibrium constant (L mg^−1^) *C*_*e*_: Ion final concentration in solution (mg L^−1^) *β*_*s*_: Heterogeneity factor

### Sorption thermodynamics

Equilibrium sorption measurements allowed determination of the thermodynamic parameters, including the thermodynamic equilibrium constant (${K}_{e}^\circ$), the standard sorption Gibbs energy ($\Delta G^\circ$), the standard sorption enthalpy ($\Delta H^\circ$), and the standard sorption entropy ($\Delta S^\circ$) by the following equations (Lima et al. 2020, 2019):

3 $${K_{e}}^{^\circ }=\frac{\left(1000\cdot {K}_{model}\cdot {M}_{sorbate}\right)\cdot {\lfloor{Sorbate}\rfloor}^{^\circ }}{\gamma }$$

4 $${K_{e}}^\circ\;=\;exp\;\left\lceil\frac{\Delta S^\circ}R\;-\;\left(\frac{\Delta H^\circ}R\right)\;\bullet\;\frac1T\right\rceil$$

5 $$\Delta G^\circ =\Delta H^\circ -T\Delta S^\circ$$

where:

*Ke*°: Thermodynamic Equilibrium Constant (dimensionless).

*K*_*model*_: Isotherm model equilibrium constant (L mg^−1^).

*M*_*sorbate*_: Molecular weight of sorbate (in this study: Cu = 63.55 g mol^−1^).

[*Sorbate*]°: Standard concentration of the sorbate (1 mol L^−1^).

γ: Coefficient of activity (unitary for dilute solutions and dimensionless).

$\Delta H^\circ$: Standard sorption enthalpy (kJ mol^−1^).

$\Delta S^\circ$: Standard sorption entropy (J.mol^−1^ K^−1^).

$\Delta G^\circ$: Standard sorption Gibbs free energy (J mol^−1^).

*T*: Absolute temperature (K).

*R*: Universal gas constant (8.3145 J K^−1^ mol^−1^).

Equation (4) represents the nonlinear form of the classical Van’t Hoff equation.

### Effect of sorbent dosage

Aliquots of 25 mL of Cu(II) solution ($\cong$ 250 mg⋅L^−1^) were added to an appropriate mass of sorbent to obtain a sorbent dosage ranging between 1 and 8 g L^−1^. The tests were carried out under the following operational conditions: buffered pH = 5.2; T = 25 °C; reaction time = 60 min; agitation speed = 100 rpm.

### Desorption and reusability tests

The desorption experiments were carried out according to the methodology reported elsewhere (Gunawardene et al. 2021) using the NaOH-W sorbent after the end of Cu(II) sorption tests. The material was dried in an oven at 50 °C for 1.5 h and then mixed with 100 mL of 0.1 M HCl solution (in duplicate) under stirring at 100 rpm and 25 °C. Finally, the liquid phase, collected at different contact times, was filtered and the concentration of the Cu(II) ions was determined by FAAS, as previously described.

The desorption efficiency (DE) was calculated according to Eq. (6) (Gunawardene et al. 2021; Santos et al. 2022), where *C*_*t*_ (mg⋅L^−1^) is the concentration of copper ions in the desorption solution at time *t* (min), *V* is volume of the desorption solution, and *m*_*0*_ (g) is the amount of Cu (II) ions sorbed in the sorption test.

6 $$DE\;(\%)=\frac{C_{t}\bullet V}{m_0}\bullet100$$

For the reusability tests, a selected sample of sorbent underwent the sorption/desorption cycle for three times under the above-mentioned conditions.

### Effect of initial pH on copper sorption

The influence of pH on sorption was evaluated in the range of pH 1–5 adding 0.1 g aliquots of sorbent to 25 mL of copper solution ($\cong$ 250 mg⋅L^−1^). The pH was corrected by adding few drops of concentrated HCl solution. The samples were kept at 25 °C under agitation of 100 rpm for 60 min.

### Effect of coexisting ions on copper sorption

The influence of coexisting ions was explored by comparing the sorption performance of three samples containing copper only, copper + NaCl 0.01 M, and copper + 0.01 M CaCl_2_, respectively. The other operational conditions were: sorbent dosage = 4 g L^−1^; initial copper concentration $\cong$ 250 mg L^−1^; buffered pH = 5.2; reaction time = 60 min; agitation speed = 100 rpm; T = 25 °C.

### Analysis error functions

Some analysis error functions, namely adjusted *R*^2^ (*R*^2^_adj_.), chi-square (χ^2^), sum of square error (SSE), and Akaike Information Criterion (AICc) (Teixeira et al. 2023) (see Table S1) were used to assess the suitability of the used sorption models.

## Results and discussion

### pH_pzc_

**Fig. 1:**
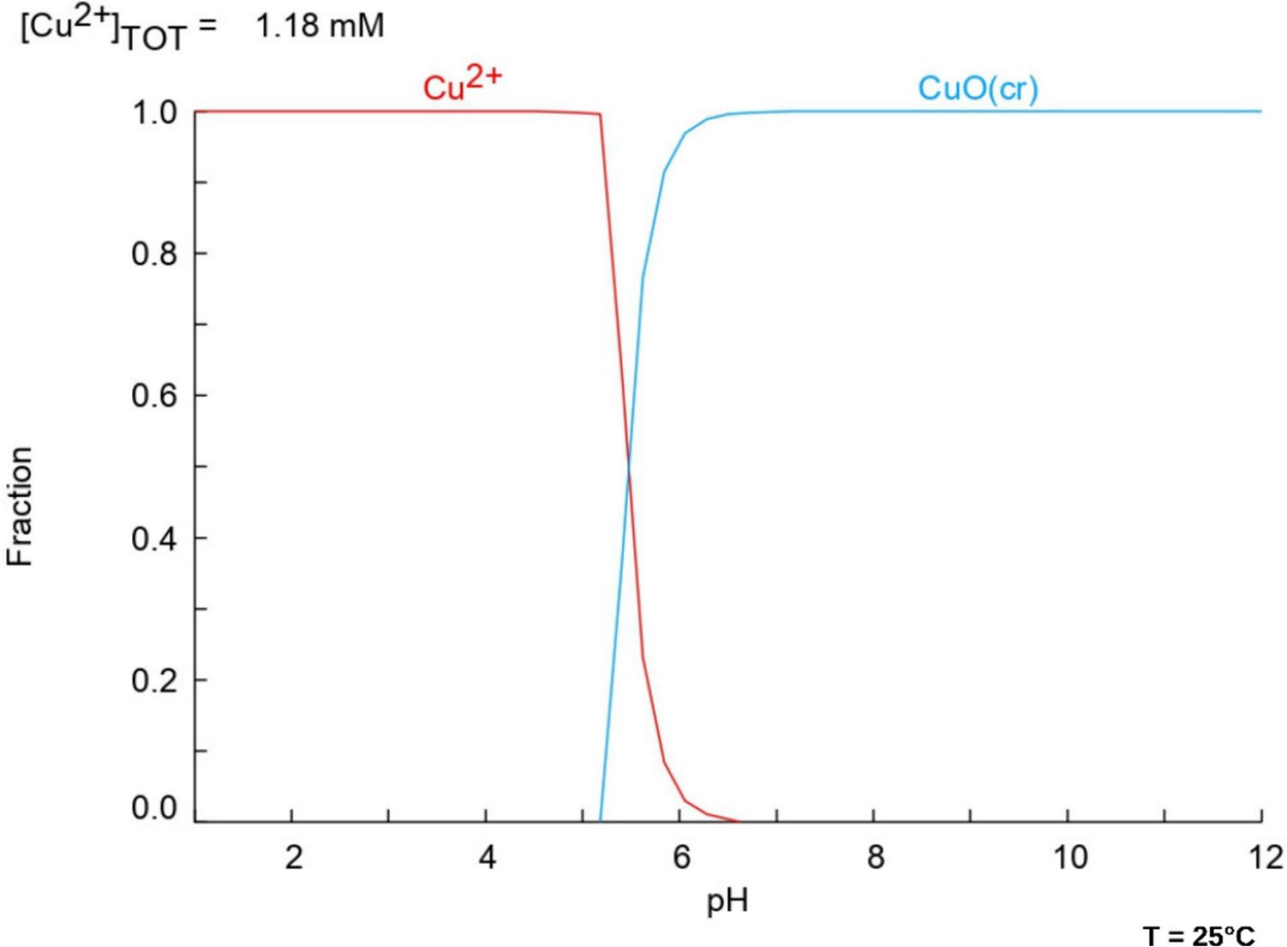
Copper speciation diagram as a function of solution pH

**Fig. 2:**
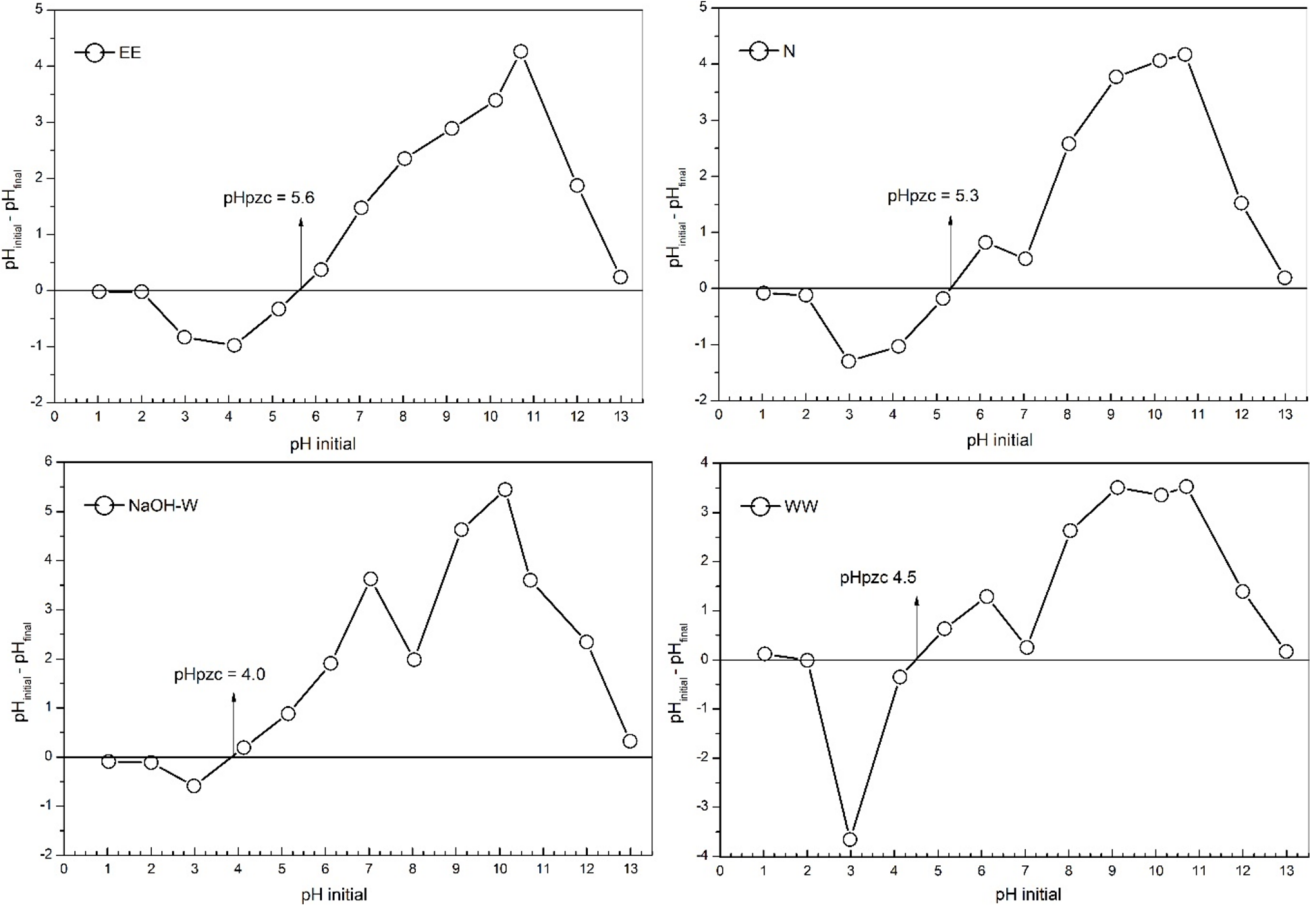
Determination of the point of zero charge (pH_pzc_) for the sorbents tested

The speciation diagram reported in Fig. 1 indicates that copper is predominantly available in its Cu^2+^ form at the experimental buffered pH (pH = 5.2). This suggest that sorption of copper could be facilitated via electrostatic attraction in the presence of sorbent exhibiting a negatively charged surface (Menezes et al. 2020). The PZC experiments (Fig. 2) reveal that at the working pH, the surface of WW and NaOH-W is characterized by a prevalence of negative charges, while natural (N) and EE sorbents are close to a neutral charge scenario. This division in the surface charge density influences the copper uptake behavior.

### SEM-EDS and FT-IR analysis

**Fig. 3:**
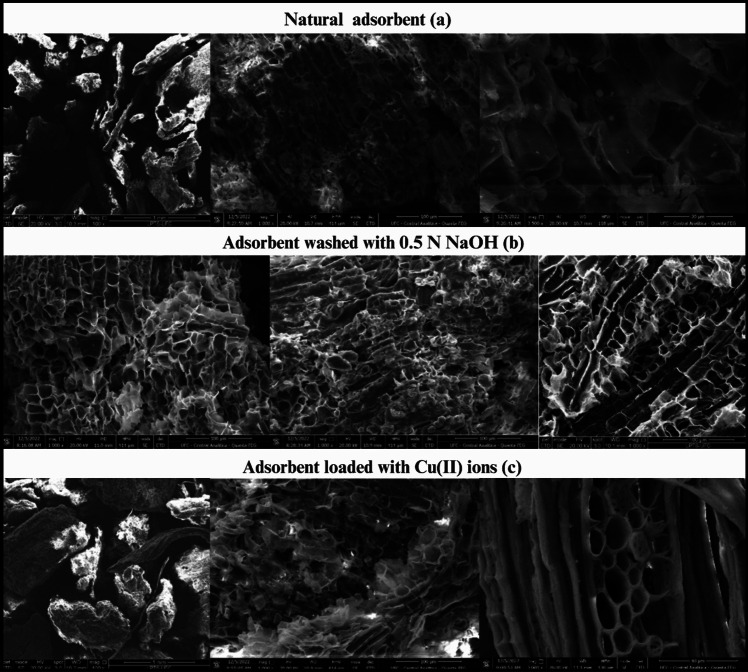
Scanning electron microscopy images of sorbent before and after contact with Cu(II) ions

**Fig. 4:**
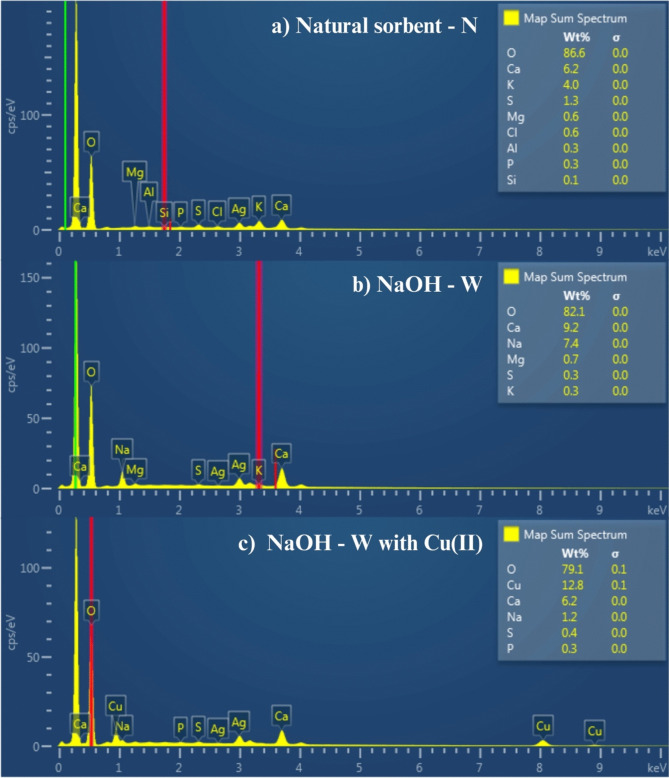
SEM–EDS compositional analysis of sorbent: **a** natural (N), **b** washed with NaOH (NaOH-W) before Cu(II) sorption and **c)** after Cu(II) sorption

The micrographs in Fig. 3 show the typical porosity and topography of the lignocellulosic material. No noticeable changes in the structure (topography) of the natural material were observed after washing with NaOH-W. However, SEM–EDS analysis reported in Fig. 4 indicates that NaOH treatment leads to an increase of Na^+^ ions in the natural sorbent (from 0.0 to 7.4%) while reducing the concentration of K^+^ ions (from 4.0 to 0.3%). This suggests the occurrence of an ion exchange process between K^+^ and Na^+^ (Fig. 4a and b).

Ion exchange might also account for the Cu(II) uptake. As can be seen from Fig. 4b and c, the sorption of Cu(II) onto NaOH-W is accompanied by a reduction in Na^+^ content from 7.4 to 1.2%.

**Fig. 5:**
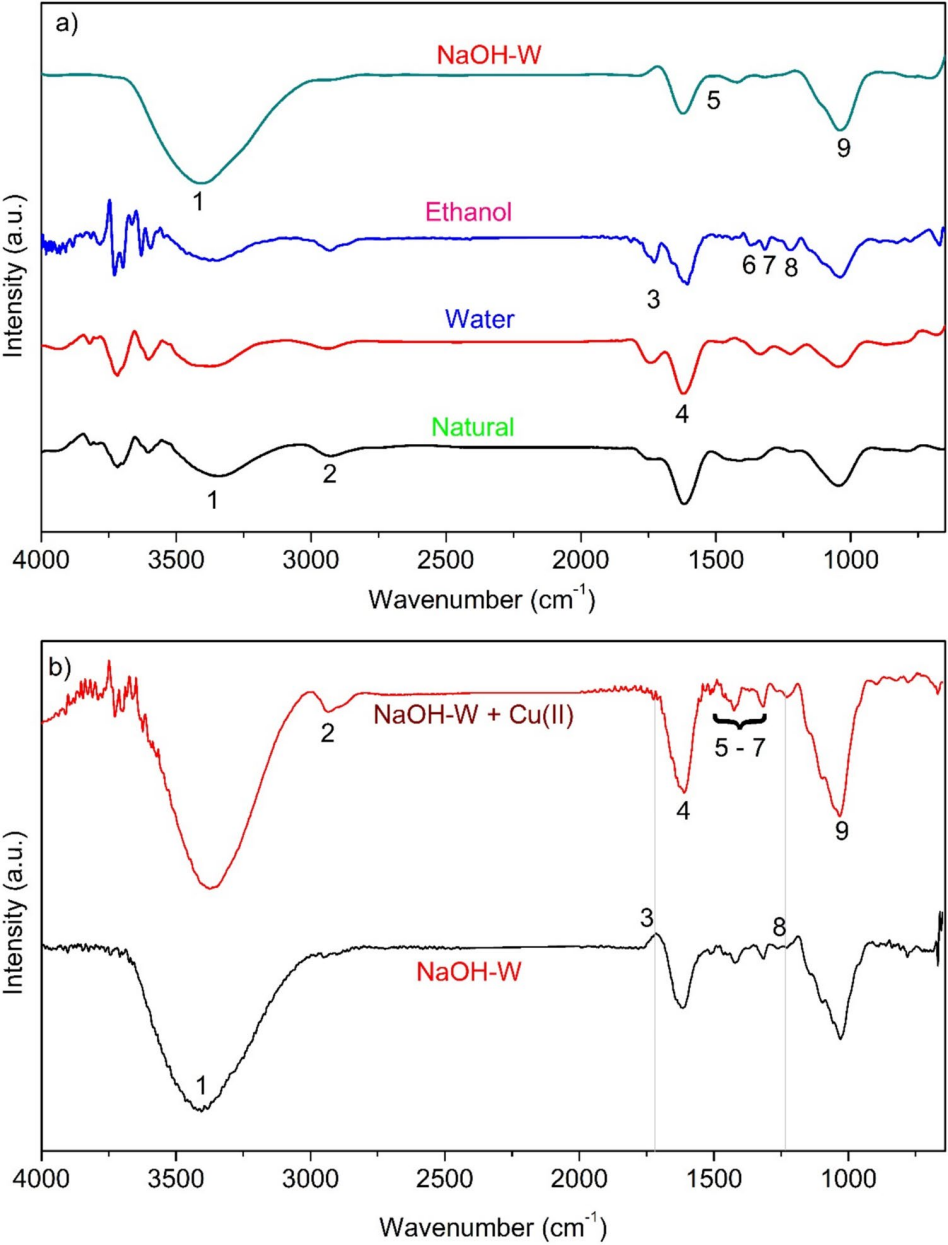
**a** FT-IR spectra of sorbent in its natural form, washed with ethanol, water and 0.5 N NaOH; **b** FT-IR spectra of NaOH-W before and after contact with Cu(II) ions. See Table S2 for peaks assignment

From the chemical point of view, Juá barks is a mixture of organic (major) and inorganic (minor) matter comprising a variety of solid and fluidwell-associated phases. The organic matter consists of non-crystalline (cellulose, lignin, hemicellulose, etc.) and crystalline (organic minerals) moieties (El-Azazy et al. 2023). The FT-IR spectra of bark samples are shown in Fig. 5a (peaks assignment is reported in Table S2).

Upon the inspection of the results of FT-IR measurements, it results that the sorbent washed with 0.5 N NaOH showed visible changes in the peaks of some functional groups. Likewise, it exhibited a higher Cu(II) sorption capacity (see sorption kinetic results below) as compared to the other sorbents. The disappearance or reduction of the peaks corresponding to functional groups 2 (− CH stretching at 2925.8 cm^−1^), 3 (C = O of carboxylic, carbonylic, acetyl groups at 1728.4 cm^−1^), 8 (C − O stretching in lignin and − COO^−^ vibration of acetyl groups in hemicellulose at 1222.2 cm^−1^), smoothing of peak 6 (− CH bending and -CH_3_ symmetrical angular vibration in cellulose and hemicellulose at 1369.1 cm^−1^), and peak 9 (C − O stretching in C(6) lignin and cellulose, and stretching in holocellulose at 1039.4 cm^−1^) suggest changes in the structures of cellulose and hemicellulose. Additionally, the intensity of peak 5, related to aromatic skeletal vibrations and C = C stretching vibrations in aromatic rings of lignin, suggests that the washing and alkaline processes partially removed them and/or disrupted their structures (Souza et al. 2020). Moreover, the evident increase in peaks 1 and 9 upon NaOH treatment points to an enrichment in oxygen-containing functional groups (Usmani et al. 2022).

Owing to its slightly higher sorptive capacity, NaOH-W sorbent was selected for the comparative analysis of the FT-IR spectra (Fig. 5-b). Upon contact with copper, noticeable changes were observed, such as the attenuation and shift to lower wavenumber of peak 1 (3396.1 cm^−1^) related to the vibrational stretching of − OH groups and the reappearance of peak 2. However, the main alterations were associated with the C = O and C − O functions of carbonyl and carboxyl groups, including the disappearance of peak 3 (C = O of carboxylic, carbonylic, acetyl groups at 1728.4 cm^−1^), and increased intensity of peak 4 (− COOH groups stretching vibration in aromatic groups associated with cellulose, lignin, and hemicellulose at 1616.1 cm^−1^) and peak 9 (1039.4 cm^−1^), indicating changes in C-O bonds, due to the interaction with copper, not only from carboxyl groups but also from alcohols. Additionally, peak 8 (− COO^−^ vibration of acetyl groups in hemicellulose) reappeared.

The bands encompassing the peaks in the range from 1511.9 to 1315.5 cm^−1^ (5 to 7, related to aromatic structures, primarily of lignin) showed little improvement in signal upon the presence of copper, indicating no significant interaction.

### Sorption kinetics

**Fig. 6:**
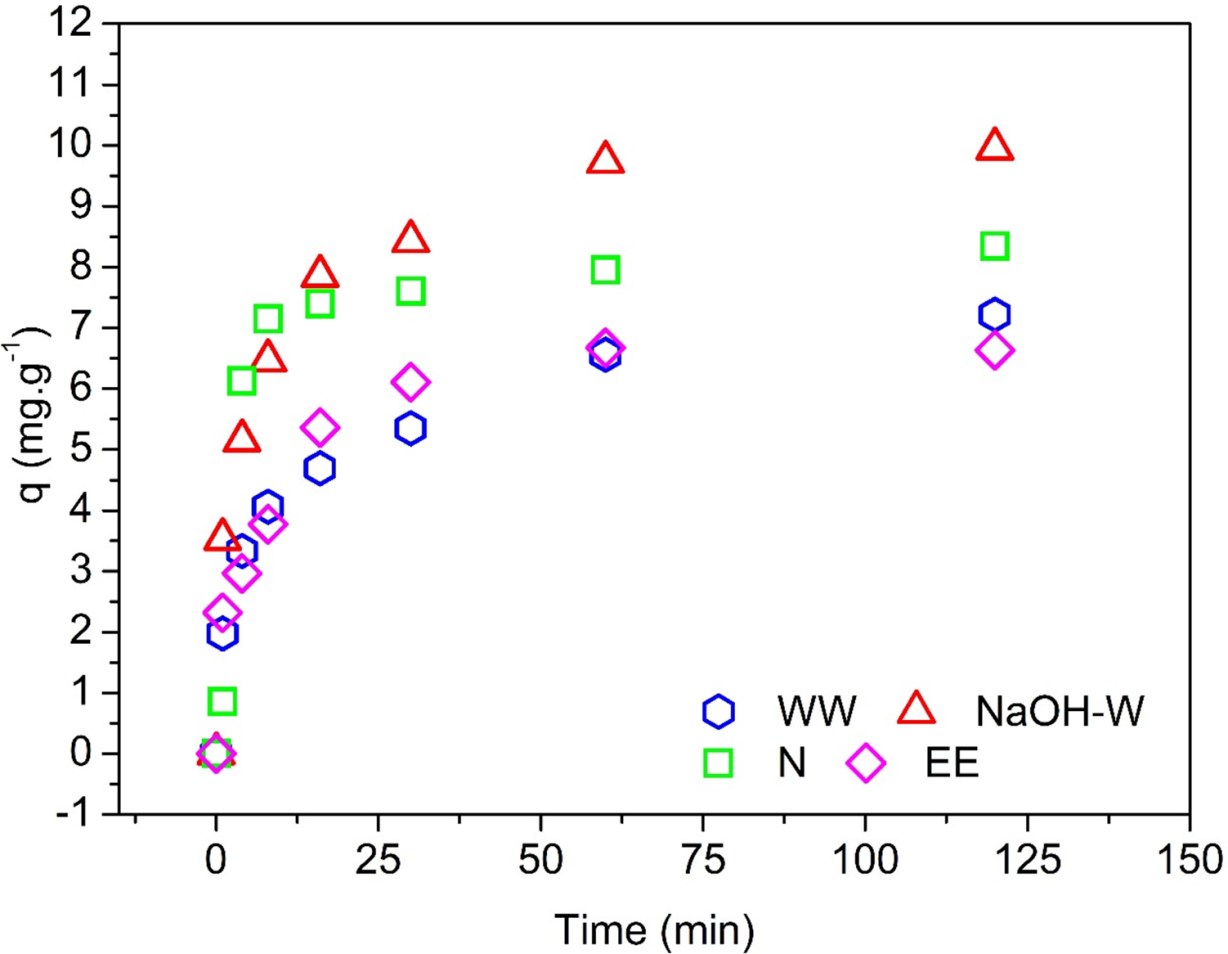
Sorption kinetics tested for the removal of Cu(II) ions under the following conditions: T = 25 °C, pH = 5.2, initial solute concentration = 75 mg L^−1^, agitation speed = 100 rpm, and mass of adsorbent = 0.1 g

All the sorbents reached equilibrium within about 2 h (Fig. 6). The maximum experimental sorption capacities are reported in Table 4; the highest (9.9 mg g^−1^) and lowest (6.0 mg g^−1^) values were achieved by the NaOH-washed and the ethanol-washed sorbent, respectively.

**Table 4:** Kinetic parameters obtained for the sorption of Cu(II)

Nonlinear PFO Model								
Sorbent	q_exp_^(*)^ (mg g^−1^)	q_model_^(*)^ (mg g^−1^)	Error relative (%)	*R*^2^ adjusted	SSE	χ^2^		k_1_ (min^−1^)
WW	7.2	6.2	14.0	0.8745	4.26	0.71		0.146
N	8.4	7.9	5.4	0.9658	2.25	0.38		0.289
EE	6.7	6.4	3.9	0.9174	2.83	0.47		0.136
NaOH-W	9.9	9.0	9.0	0.9064	6.47	1.08		0.201
Nonlinear PSO Model								
Sorbent	q_exp_^(*)^ (mg g^−1^)	q_model_^(*)^ (mg g^−1^)	Error relative (%)	*R*^2^ adjusted	SSE	χ^2^		k_2_ (g mg^−1^ min^−1^)
WW	7.2	6.9	4.8	0.9467	1.81	0.30		0.029
N	8.4	8.6	3.0	0.9422	3.81	0.63		0.045
EE	6.7	6.9	4.2	0.9467	1.69	0.28		0.030
NaOH-W	9.9	9.8	1.7	0.9650	2.42	0.40		0.032
Nonlinear Elovich Model								
Sorbent	q_exp_^(*)^ (mg g^−1^)	q_model_^(*)^ (mg g^−1^)	Error relative (%)	*R*^2^ adjusted	SSE	χ^2^	β_E_ (g mg^−1^)	α (mg g^−1^ min^−1^)
WW	7.2	7.2	0.8	0.9964	0.12	0.02	0.86	4.7
N	8.4	9.3	11.7	0.8422	10.39	1.73	0.75	12.5
EE	6.7	7.1	7.1	0.9639	1.23	0.21	0.91	6.1
NaOH-W	9.9	10.4	4.6	0.9917	0.58	0.10	0.68	15.0

^(*)^q_exp_ and q_model_ are the equilibrium sorptive capacity obtained experimentally and predicted by the model, respectively

Table 4 shows the results of the curve fitting analysis for the kinetic data reported in Fig. 6 by using the PFO, PSO, and Elovich models. The higher values of *R*^2^_adj_ and the lower error function values for all tested sorbents (with the exception of N sample data which slightly better conform to the PFO model) suggest that the PSO model adequately describes the Cu(II) sorption kinetics, i.e., that the rate of sorption is proportional to the square of the distance from the equilibrium (Salvestrini 2018).

It is important to note that the PFO and PSO model are pure descriptive (empirical) models; they may help in predicting the temporal evolution of the sorbate but do not provide any information on the sorption mechanism.

To shed light on the sorption mechanism, we used the Boyd’s model (Boyd et al. 1947; Reichenberg 1953). It has a theoretical background that relies on the assumption that the rate of sorption is controlled by diffusion phenomena. In particular, the Boyd model may indicate whether the rate-limiting step of sorption is the movement of the solute through the stagnant film around the external surface of the sorbent (known as external or film diffusion) or within the sorbent particle (intraparticle diffusion, which may be due to pore diffusion, surface diffusion, or a combination of both (Yao and Chen 2017). The reliability of the Boyd model can be assessed from the linearity of the plot of *Bt vs t*. More specifically, if the straight line passes from the origin, it is assumed that for the evaluated time range, the main resistance to mass transfer is the intraparticle diffusion. If the intercept with the y-axis differs from zero, the sorption kinetics is governed by film or by both film and intraparticle diffusion (Al-Muhtaseb et al. 2011; Viegas et al. 2014; Iravani Mohammadabadi and Javanbakht 2021).

The relative magnitude of film and intraparticle mass transfer resistances depends on sorption conditions, such as stirring speed and sorbent size. High stirring speed can reduce or even eliminate film mass transfer resistance. Smaller particle size usually results in slower intraparticle diffusion resistance (Yao and Chen 2017).

**Fig. 7:**
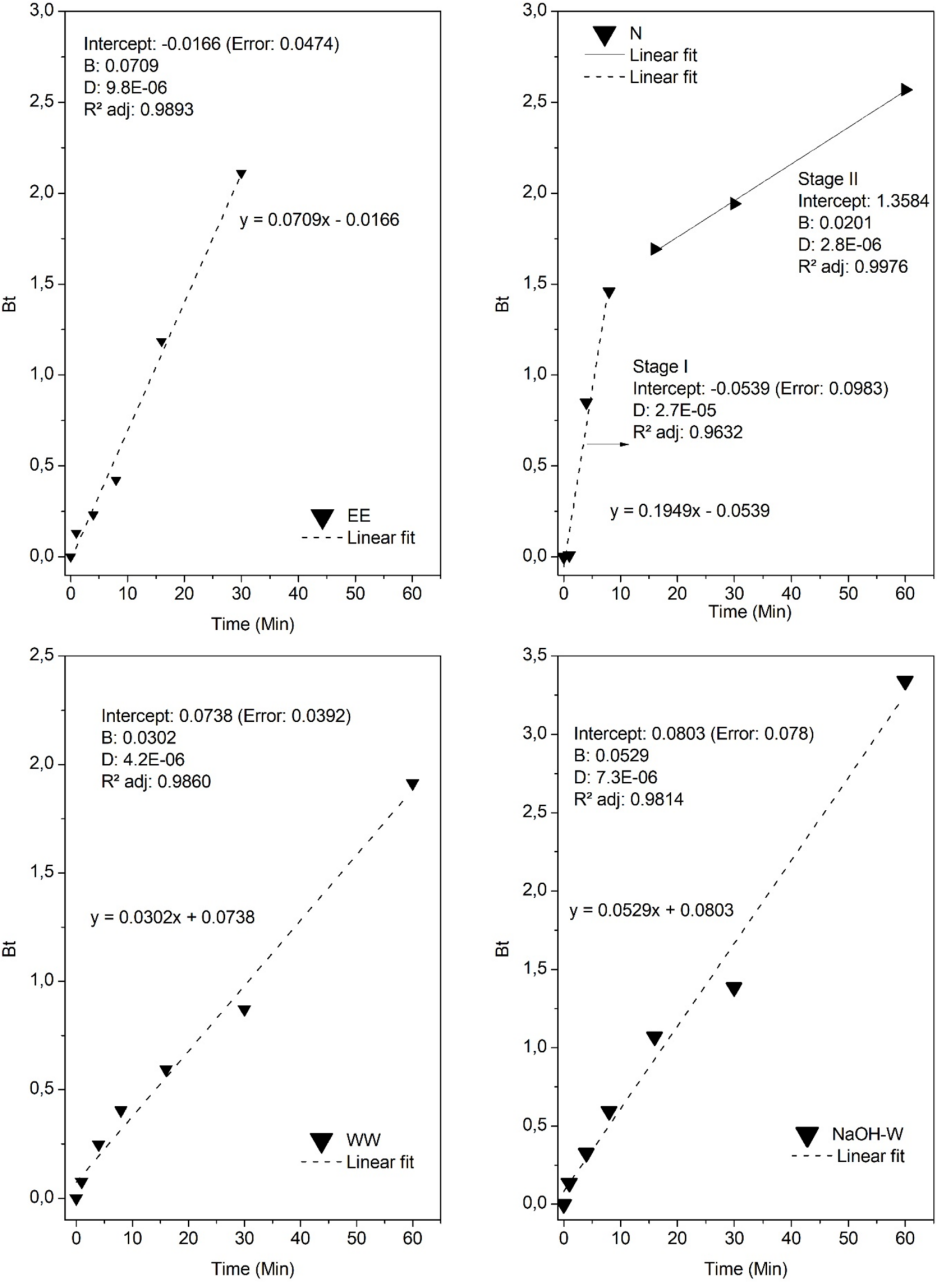
Application of Boyd’s intraparticle and external diffusion model for the sorption kinetic data of Cu(II); B = Boyd model constant (determined by the slope of the straight line) and D = Boyd effective diffusion coefficient (cm^2^ min)

From the analysis of the graphs in Fig. 7, it can be observed that the intercepts of the straight lines for all the sorbents are approximately equal to zero, (see the standard error of the intercepts reported in Fig. 7). This finding suggests the predominant role of intraparticle diffusion in controlling the uptake of sorbate up to 8 min of contact time for N sorbent, and for the whole sorption process in the case of the other sorbents.

The sorption experimental data of the sorbent materials that underwent washing (WW, NaOH-W, and EE) showed better fit than the natural state material (N) (see R^2^_adj_ values in Fig. 7). Moreover, they exhibit values of film and intraparticle mass transfer resistance of the same magnitude order, as well as similar shape curve throughout the contact time. This scenario implies that copper diffusion is slower in the interstices of the sorbent until 1 h, and that the applied agitation was sufficient to overcome the external resistance of the film.

### Sorption isotherms

The best performant sorbent in the kinetic experiments, NaOH-W, and, for comparison purposes, the water-washed sample (WW), was selected for investigating the sorption of Cu(II) at equilibrium.

**Fig. 8:**
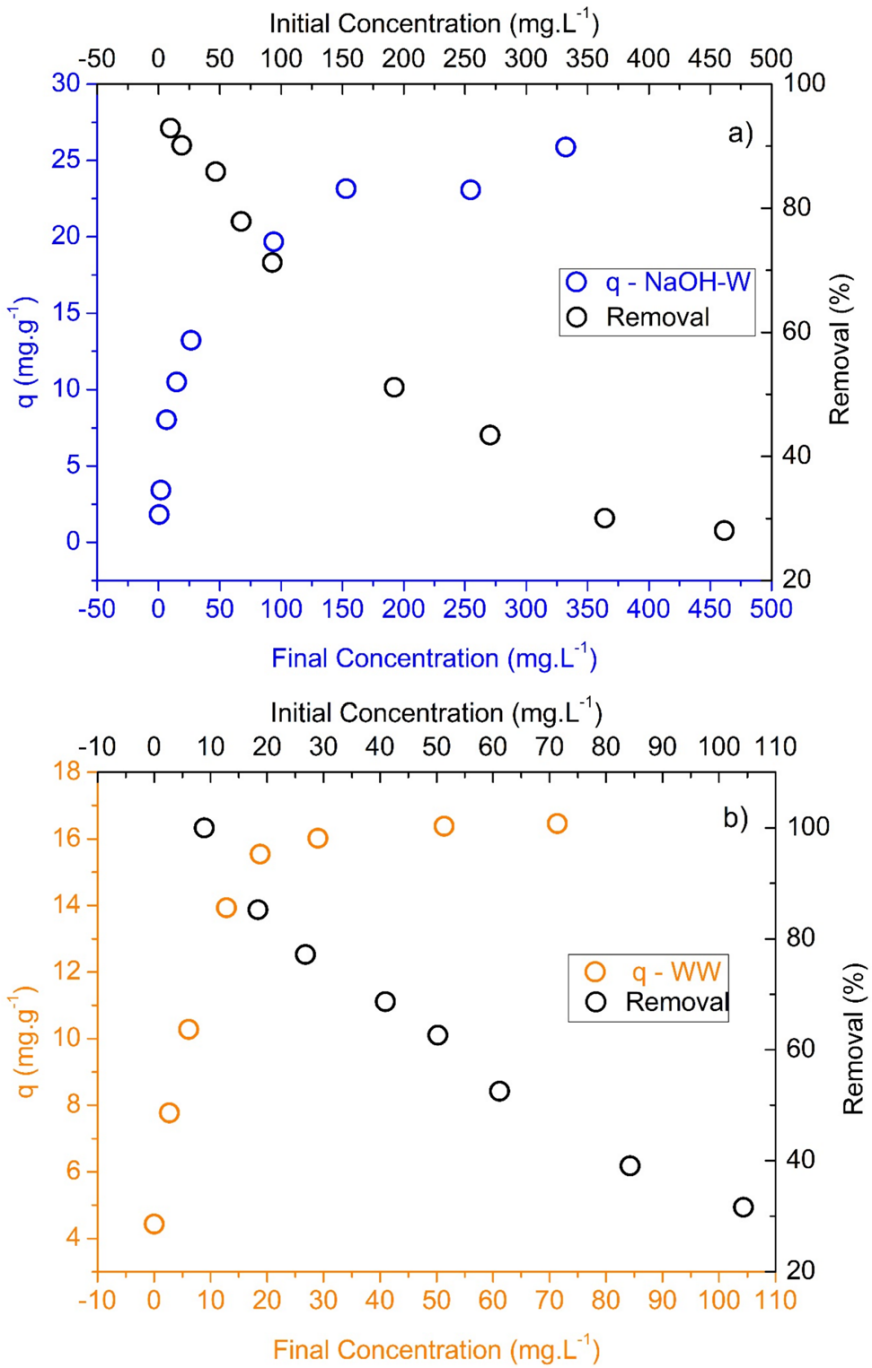
Equilibrium isotherm and removal percentage of Cu(II) ions in the presence of a) NaOH-W and b) WW. Test conditions: T = 25 °C; pH = 5.2; reaction time = 60 min; agitation speed = 100 rpm; mass of sorbent = 0.05 g

Figure 8 shows the isotherm data and the removal efficiency (%) of NaOH-W and WW towards Cu(II). As can be seen, NaOH-W exhibits a higher affinity for Cu(II); the maximum sorption capacities of NaOH-W and WW are 25.9 mg g^−1^ and 16.5 mg g^−1^, respectively.

The isotherm data were fitted by different models and the results of the curve fitting procedure are reported in Table 5, Table S4 and Fig. S2. Based on the results, NaOH-W and WW isotherms are better described by the Sips and the Langmuir model, respectively.

**Table 5:** Sorption equilibrium parameters for Cu(II) sorption onto NaOH-W and WW (Fig. 8)

Model			R^2^ adjusted	SSE	χ^2^	AICc
	Parameters – NaOH-W sorbent					
	*q*_*max*_ *. experimental* (mg·g^−1^)	25.9				
Langmuir	*q*_*max*_ (mg.g^−1^)	25.8	0.9770	13.07	1.87	11.62
	*K*_*L*_ (L.mg^−1^)	0.048				
Freundlich	*K*_*F*_ (mg g^−1^)(L.mg^−1^)^1/n^	4.371	0.9655	19.58	2.80	15.26
	*n*	3.194				
	1/*n*	0.313				
Temkin II	*K*_*T*_ (L mg^−1^)	1.361	0.9758	13.74	1.96	12.07
	*q*_*T*_ (mg g^−1^)	4.89				
Sips	*q*_*max*_ (mg g^−1^)	32.1	0.9925	3.65	0.61	6.33
	*K*_*S*_ (L mg^−1^)	0.087				
	*β*_*S*_ (dimensionless)	0.648				
	Parameters – WW sorbent					
	*q*_*max*_ *. experimental* (mg.g^−1^)	16.5				
Langmuir	*q*_*max*_ (mg g^−1^)	17.8	0.8318	21.16	3.53	16.48
	*K*_*L*_ (L mg^−1^)	0.270				
Freundlich	*K*_*F*_ (mg g^−1^)(L mg^−1^)^1/n^	7.726	0.7558	30.72	5.12	19.47
	*n*	5.088				
	1/*n*	0.196				
Temkin II	*K*_*T*_ (L mg^−1^)	7.878	0.7893	26.50	4.42	18.28
	*q*_*T*_ ( mg g^−1^)	2.82				
Sips	*q*_*max*_ (mg g^−1^)	17.5	0.7987	21.10	4.22	23.52
	*K*_*S*_ (L mg^−1^)	0.249				
	*β*_*S*_ (dimensionless)	1.073				

The Sips semi-empirical model is a versatile three-parameter isotherm that predicts the heterogeneity of the localized sorption system without sorbate-sorbate interactions. It can simulate the behavior of the Freundlich model at low sorbate concentration and that of the Langmuir isotherm at high sorbate concentration predicting the attainment of a plateau (monolayer saturation). Values of *β*_*S*_ close to or equal to 1 indicate a solid with relatively homogeneous binding sites (approaching the conditions of the Langmuir equation); otherwise, when *β*_*S*_ → 0, it reduces to the Freundlich equation, and therefore, the sorbent has heterogeneous characteristics (Ho et al. 2002; Jeppu and Clement 2012; Saadi et al. 2015; Al-Ghouti and Da’ana 2020; Chu et al. 2022; de Vargas Brião et al. 2023).

The good fit observed for the NaOH-W sorbent using the Sips model associated with a value of *β*_*S*_ significantly lower than one (0.61) suggests that the sorbent exhibits a limited number of heterogeneous sorption sites.

The isotherm data for the WW sorbent showed a better fit to the Langmuir model (*R*^2^_adj_ 0.8318 and AICc 16.48), indicating that the sorbent might consist of energetically identical and equivalent localized sites (Salvestrini 2019; Al-Ghouti and Da’ana 2020).

The difference in sorption capacity between WW and NaOH-W sorbents supports the hypothesis that NaOH treatment and Na^+^ exchange has a relevant role in the sorption process.

Table 6 shows that WW and NaOH-W exhibit a good sorption capacity for Cu(II) as compared to many sorbents derived from other vegetables, with the exception of those from the genus Ziziphus (seeds) obtained with more advanced treatments such as ultrasound or carbonization. However, it should be noted that such advanced treatments are more expensive and difficult to implement as compared to that used for preparing WW and NaOH-W sorbents.

**Table 6:** Maximum sorption capacity of ions Cu(II) ions by sorbents derived from Ziziphus genero and other vegetables

Species/Type	Part	Modifications	Experimental Conditions			Sorption capacity		Reference
			pH	°C	Time (min)	Langmuir (mg.g^−1^)	*R*^2^	
*Jujube*	Dried Fruit—Beads	Alginate—drying—freezing	-	-	-	20.33	0.9905	(An et al. 2015)
*Jujube*	Seed powder	Sodium alginate gel and CaCl_2_	-	30	-	3.64	0.93	(Choi et al.. 2012)
*Jujube*	Seed	Ultrasound	5	30	60	259	-	(Gayathri et al.. 2019)
*Zizyphus jujuba*	Seed shell	Activated nano porous carbon	6.5	30	60	141.07	-	(Veeravelan et al. 2016)
Wooden Sawdust	Cherry, poplar, hornbeam, andspruce sawdust	Poplar Cherry Spruce Hornbeam NaOH:	4	22	1440 No stirring	3.97 2.98 2.13 4.32 8.20 7.38 9.02 7.71	0.99 0.87 0.88 0.94 0.96 0.98 0.87 0.80	(Kovacova et al. 2020)
		Poplar Cherry Spruce Hornbeam						
Bio sorbents	Corn cob and Strychnospotatorum seed powder	No modification	5	40	60	6.24 (Experimental)	-	(Pavan Kumar et al. 2018)
Agricultural solid waste by-produc	Dried sugar beet pulp	No modification	4	25	1440	28.5 (Experimental)	-	(Aksu and Işoǧlu 2005)(
Agricultural waste	Banana leaves	No Modification Activated carbon	5	40	40 60	48.7 66.2	0.999 0.999	(Darweesh et al. 2022)
Chinese chestnuts	Shells	Steam exploded at 1.3 MPa	5	30	1440	10.86	0.993	(Qie et al. 2018)
A. indica (Neem)	Seeds	No modification 0.1 M NaOH	5.5	25	80	11.54 11.41	0.999 0.999	(Costa et al. 2020)
*Caryocar Coriaceum* Wittm	Peel	No modification	5.5	25	120	26.9	0.986	(Coelho Menezes et al. 2021)
*Z. joazeiro*	Bark	Washed by NaOH Washed by water	5.2	25	60	25.8 31.1 (Sips) 17.8	0.977 0.992 (Sips) 0.832	*This study*

### Sorption thermodynamics

**Fig. 9:**
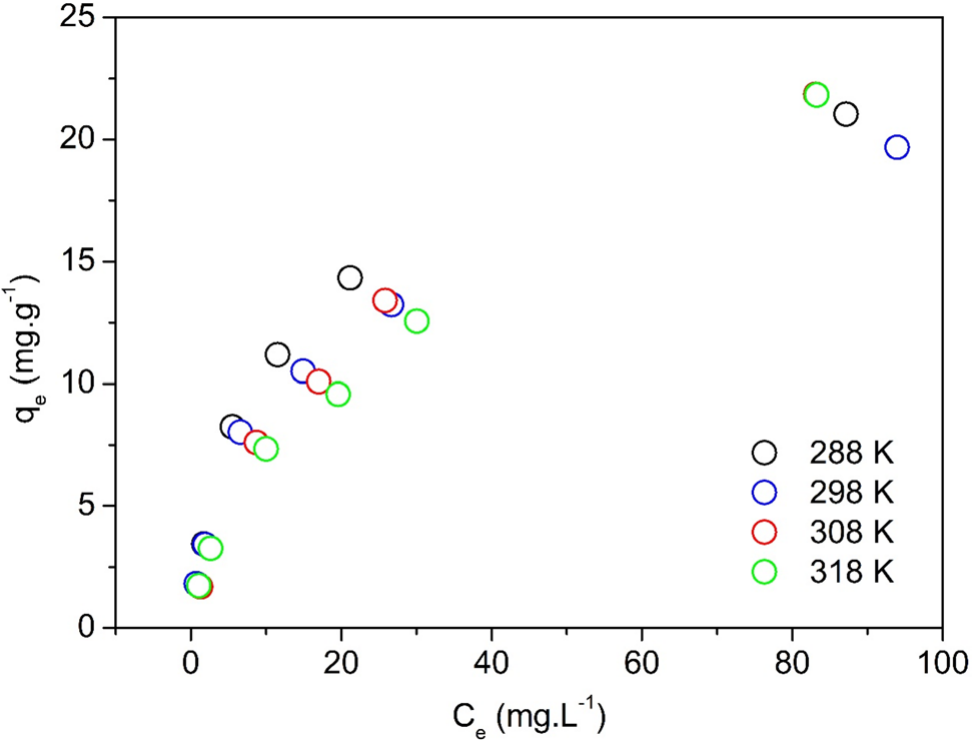
Equilibrium isotherms of Cu(II) ions sorption onto NaOH-W at different temperatures. Test conditions: T = 15–45 °C; pH = 5.2; reaction time = 60 min; agitation speed 100 rpm; mass of sorbent = 0.05 g; liquid volume = 10 mL; initial solute concentration = 10—192 mg L.^−1^

**Fig. 10:**
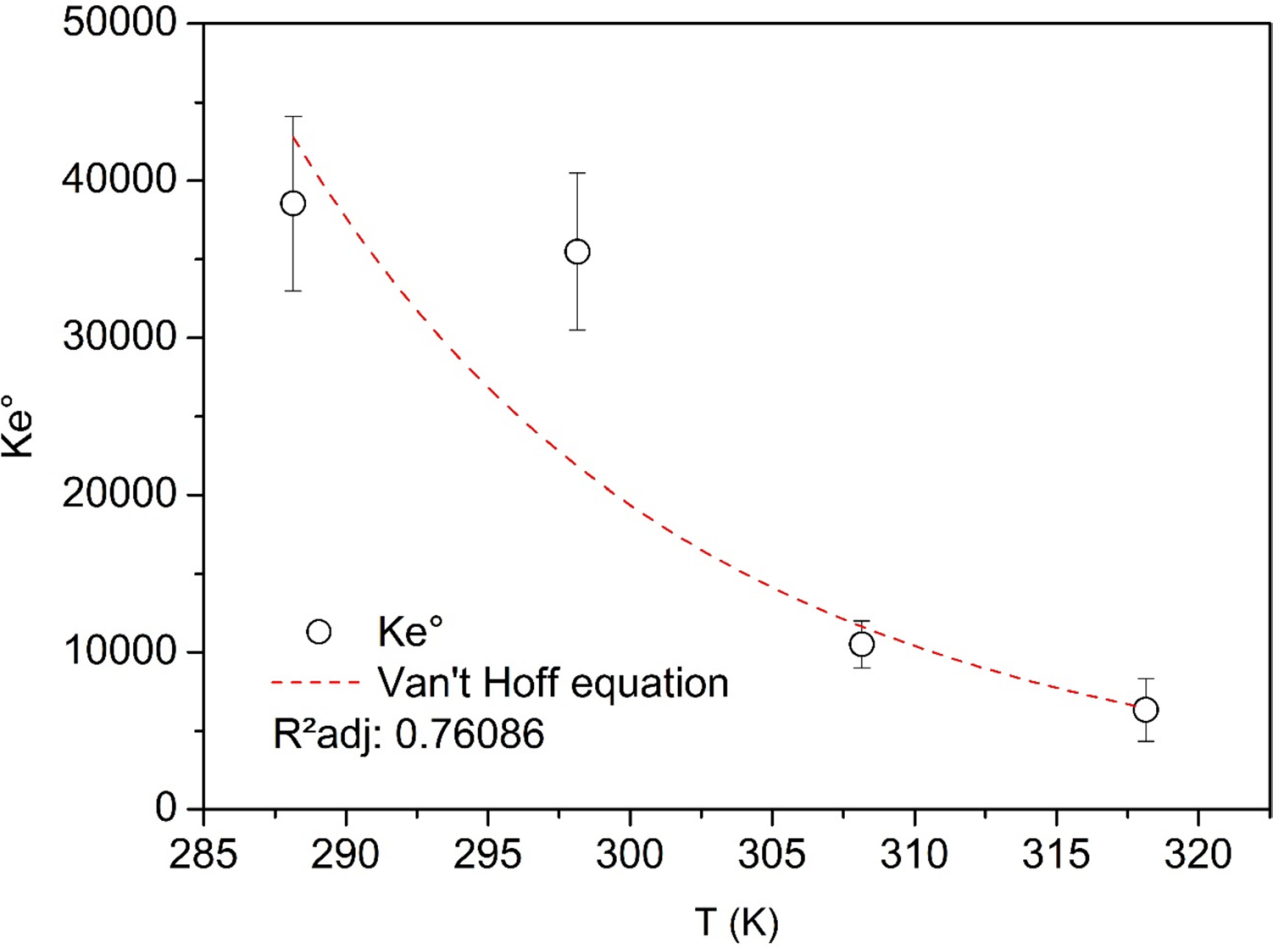
Dependence of the thermodynamic equilibrium constant on temperature. The curve in figure was obtained by non-linear curve fitting of the data using the Van’t Hoff equation

The effect of temperature on the sorption isotherms of Cu(II) onto NaOH-W is shown in Fig. 9. Among the applied isotherm models, the Temkin II model better described the change of Cu(II) sorption with temperature, as inferred by the lowest AICc value (Table 7, Table S5 and Fig. S3). For this reason, the Temkin II equilibrium constants were used in the Van't Hoff equation to determine the sorption thermodynamic parameters (Fig. 10). The results indicate that the sorption of Cu(II) is an exothermic process (Δ*H*° =  − 48.1 ± 13.5 kJ.mol^−1^) (Table 8).

**Table 7:** Equilibrium isotherm parameters for the sorption of Cu(II) onto NaOH-W at different temperatures

Langmuir model				
Parameters / Temp.(K)	288.15	298.15	308.15	318.15
*q*_*max*_ (mg g^−1^)	23.43	21.77	28.96983	32.82
*K*_*L*_ (L mg^−1^)	0.085	0.072	0.035	0.023
*R*^*2*^ adjusted	0.9917	0.9733	0.9894	0.9710
SSE	1.701	4.623	2.299	6.146
χ^2^	0.425	1.156	0.575	1.536
AICc	2.87	8.87	4.68	10.58
Freundlich model				
*K*_*F*_ (mg g^−1^)(L mg^−1^)^1/n^	3.952	3.503	2.426	1.894
*n*	2.60	2.59	1.99	1.89
1/*n*	0.38	0.39	0.50	0.53
*R*^*2*^ adjusted	0.9454	0.9745	0.9862	0.9964
SSE	11.176	4.419	3.000	0.773
χ^2^	2.79404	1.10467	0.74989	0.19334
AICc	14.16	8.60	6.27	-1.86
Temkin II model				
*K*_*T*_ (L mg^−1^)	0.607	0.558	0.165	0.100
*q*_*T*_ (mg g^−1^)	5.34	4.89	8.06	9.58
*R*^*2*^ adjusted	0.9959	0.9966	0.9962	0.9834
SSE	0.835	0.596	0.836	3.530
χ^2^	0.209	0.149	0.209	0.882
AICc	-1.40	-3.42	-1.39	7.25
Sips model				
*q*_*max*_ (mg g^−1^)	25.90	30.09	40.38	N.C.*
*K*_*S*_ (L mg^−1^)	0.100	0.092	0.042	0.003
*b*	0.08	0.07	0.75	0.56
*R*^*2*^ adjusted	0.9955	0.9944	0.9964	0.9973
SSE	0.697	0.727	0.590	0.426
χ^2^	0.232	0.242	0.197	0.142
AICc	9.25	9.50	8.24	6.29

* N.C. = *no convergence*

**Table 8:** Thermodynamic parameters for the sorption of Cu(II) onto NaOH-W sorbent

T (K)	∆*G*° (kJ mol^−1^)	∆*H*° (kJ mol^−1^)	∆*S*° (kJ mol^−1^ K^−1^)
288.15	− 25.55 ± 1.40	− 48.1 ± 13.5	− 0.0781 ± 0.04213
298.15	− 24.77 ± 0.98		
308.15	− 23.98 ± 0.56		
318.15	− 23.20 ± 0.13		

The values of Δ*G*° (Table 8) are negative and increase with the temperature, indicating that the sorption process is favorable at lower temperatures (Fenti et al. 2019; Salvestrini et al. 2022, 2014; Salvestrini and Bollinger 2022).

### Effect of the presence of saponins on copper sorption

Triterpene saponins are important constituents of *Z. joazeiro* bark, accounting for 2 to 10% of its composition (Ribeiro et al. 2014). The most common triterpene saponins found in this species are Jujuboside B or B1 (isomer), Jujuboside, medicoside C, orbacopasaponin C, and 3-O-[β-D-Glucopyranosyl (1 → 2)α-L-arabinopyranosyl]-20-O-α-L-rhamnopyranosyl-jujubogenin (do Nascimento et al. 2020). Owing to their chemical nature (e.g., presence of carboxylic and − OH groups) saponins could be able to interact with copper ions.

However, the Afrosimetric Index revealed the following ranking for the highest saponins content: Natural > Water > NaOH-W > Ethanol (see supplementary materials for details – Table S3). The present data suggest that the saponins content does not significantly affect the sorption capacity of the sorbents; however, the effective participation of saponins in the copper uptake can neither be confirmed nor ruled out.

### Effect of sorbent dosage

**Fig. 11:**
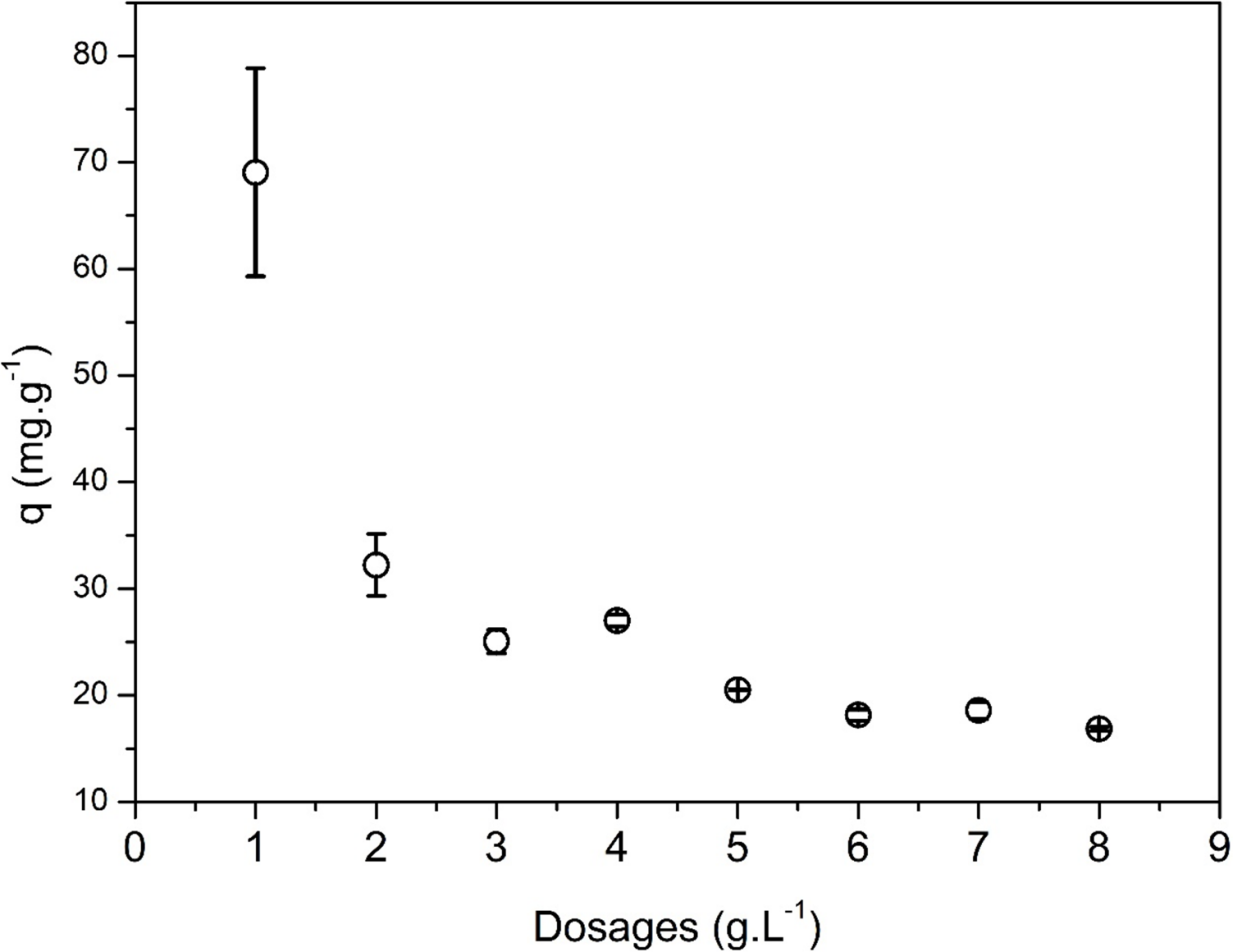
Effect of the sorbent dosage on the sorption of copper. Test conditions: T = 25 °C; pH = 5.2; reaction time = 60 min; agitation speed 100 rpm; mass of sorbent = 0.025 – 0.2 g; liquid volume = 25 mL; initial solute concentration = 253 mg L.^−1^

Figure 11 shows the sorption of copper as affected by the sorbent dosage. In line with other studies (Salman et al. 2023), the amount of copper sorbed per gram of sorbent decreases with the increase of sorbent dosage because of the increasing availability of sorption sites at a fixed initial concentration of the solute and thus suggesting the absence of detrimental effect (e.g., aggregation) on the sorbent performances. The sorbed amount markedly drops passing from 1 to 2 g L^−1^ of sorbent dosage and does not change significantly for sorbent dosage values higher than 4 g L^−1^. The removal efficiency of copper from solution increases from about 27 to 53% with increasing the sorbent dosage from 1 to 8 g L^−1^, respectively.

### Reusability tests

**Fig. 12:**
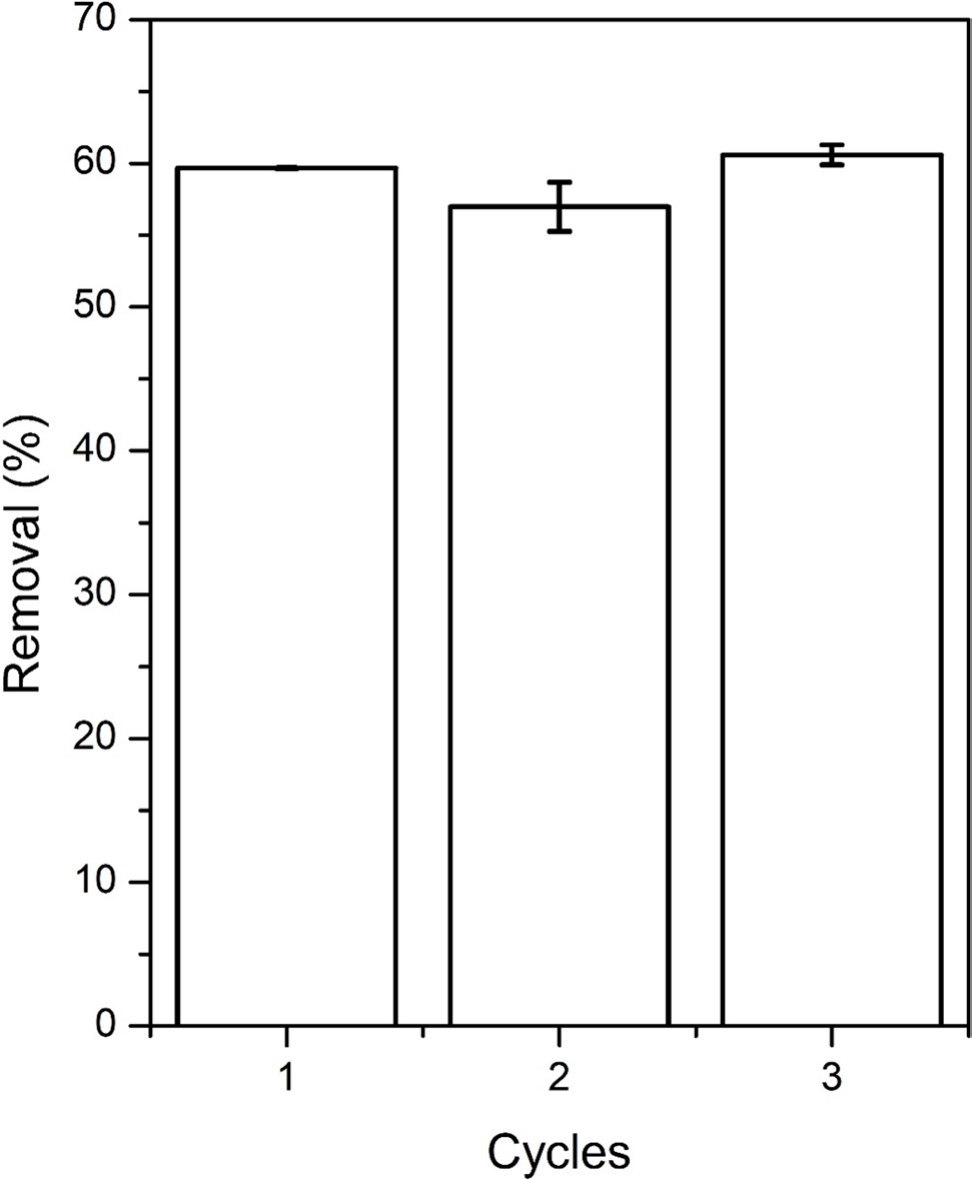
Copper ion removal efficiency after sorption/desorption cycles. Sorption test conditions: T = 25 °C; pH = 5.2; reaction time = 60 min; agitation speed 100 rpm; mass of sorbent = 0.1 g; liquid volume = 25 mL; initial solute concentration = 253 mg L^−1^. Desorption test conditions: T = 25 °C; reaction time = 10 min; agitation speed 100 rpm; liquid volume = 100 mL HCl 0.1 M

In order to evaluate the reusability of the sorbent, three sorption–desorption tests were carried according to the experimental conditions summarized in Fig. 12. As can be noted from the figure, the removal efficiency is practically the same after three sorption/desorption cycles indicating the reusability of the sorbent material and that sorption of copper is to a large extent reversible.

### Effect of pH

**Fig. 13:**
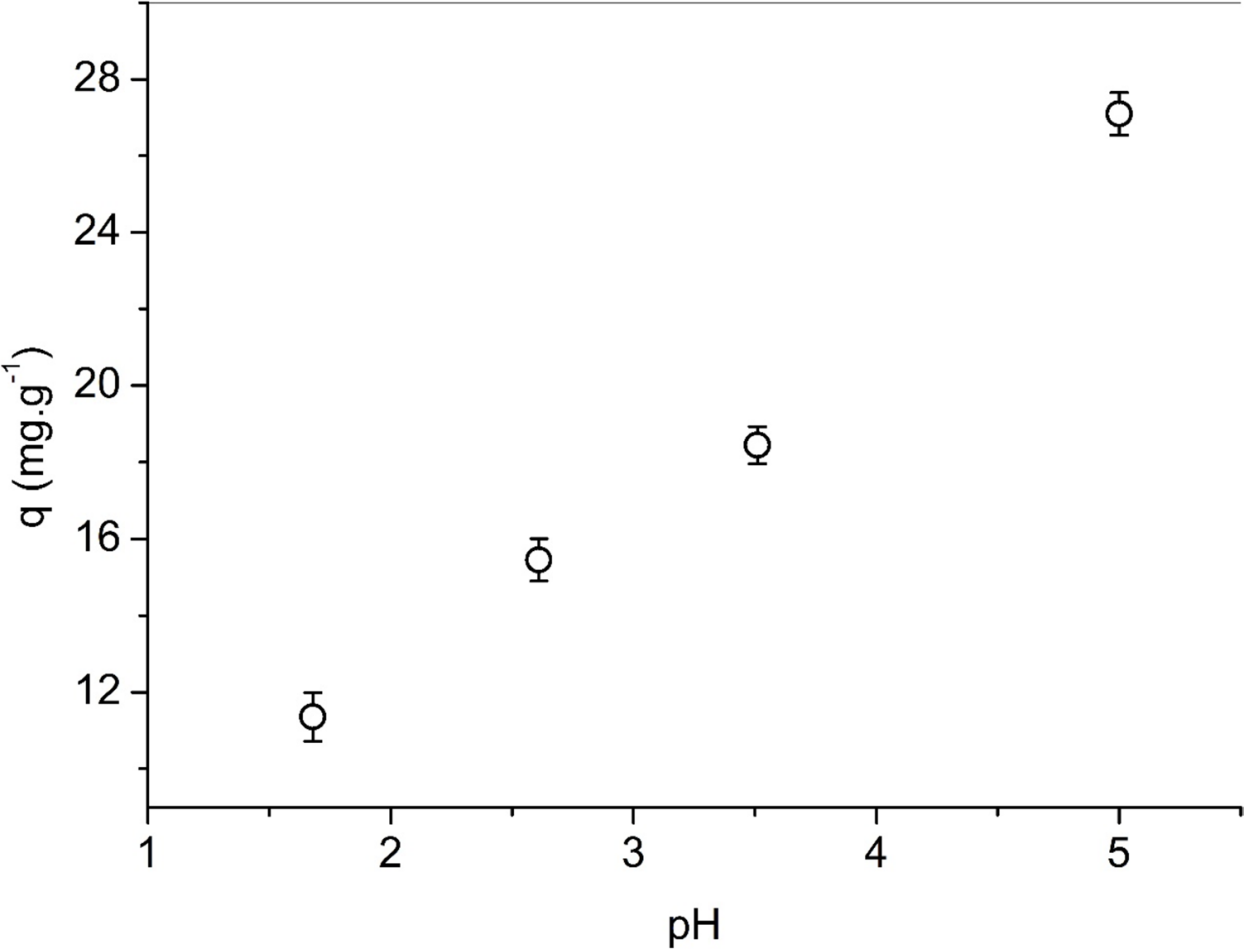
Effect of initial pH on the sorption of copper. Test conditions: T = 25 °C; pH = 1.68—5.0; reaction time = 60 min; agitation speed 100 rpm; mass of sorbent = 0.1 g; liquid volume = 25 mL; initial solute concentration = 253 mg L.^−1^

Figure 13 shows the effect of initial pH on the sorption of copper onto NaOH-W sorbent. The trend depicted in figure is the typical one reported in literature for the sorption of copper (Katiyar et al. 2021; Mariska et al. 2024).

The copper uptake decreases with the decrease of pH. This can be ascribed to the fact that at low pH, the net surface charge of the sorbent is positive due to the protonation of its carboxylic and other oxygenated groups. In such acidic conditions, electrostatic repulsion and ion exchange competition between copper and protons are responsible for the diminishing copper uptake. With the increase of pH, the sorbent surface progressively becomes more negatively charged thus promoting electrostatic interactions with copper ions. Jiang et al. (Jiang et al. 2016) reported a decrease in copper sorption at 5.0 pH with respect to 4.0 pH, possibly due to the formation of hydroxo Cu(OH)^+^ specie that might hinder sorption of copper in Cu^2+^ form. Based on the results reported in Fig. 13, this phenomenon can be neglected or excluded in the present work.

### Effect of coesisting ions

**Fig. 14:**
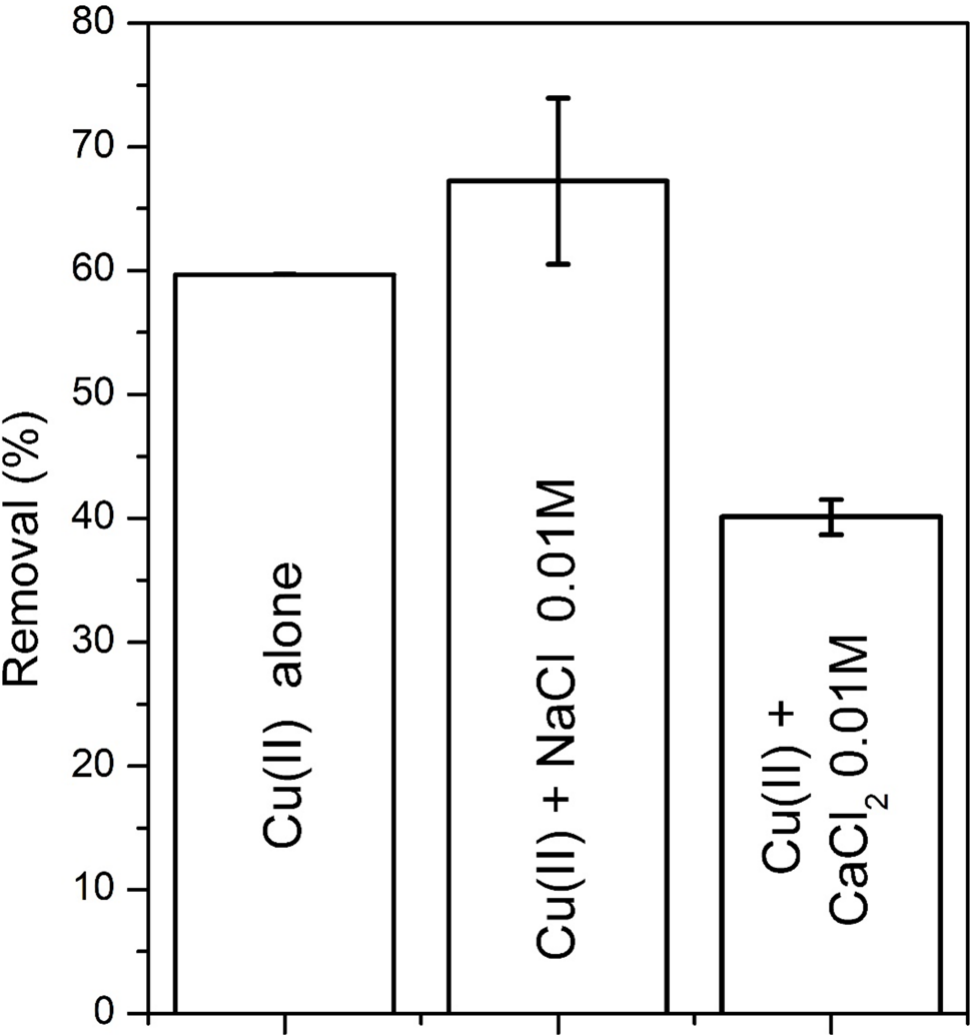
Effect of the presence of sodium and calcium ions on the sorption of copper. Test conditions: T = 25 °C; pH = 5.2; reaction time = 60 min; agitation speed 100 rpm; mass of sorbent = 0.1 g; liquid volume = 25 mL; initial solute concentration Cu(II) = 253 mg L.^−1^

Natural occurring ions in water such as sodium and calcium might compete with copper for the sorbent sites (Yang and Jiang 2014). The effect on copper sorption of the presence of sodium and calcium ions at moderately high concentration found in river water, 0.01 M (Priyadarshi 2004),is shown in Fig. 14.

The results indicate that Na^+^ has a positive effect on Cu(II) sorption: the removal efficiency of copper slightly increased from 60 to 64% after the addition of Na^+^. In contrast, the removal efficiency of copper in the presence of calcium markedly decreased to 40%. These results, in agreement with the SEM/EDS measurements, indicate that Cu(II) can more easily replace Na^+^ than Ca^2+^ on the cation exchange surface of the sorbent probably as a result of their different ionic radius and hydration energy (Teutli-Sequeira et al. 2009).

### Sorption mechanism

Based on the results collected, it is expected that multiple processes may occur concurrently during the contact of Cu(II) ions with the sorbent (NaOH-W).

The moderate low sorption enthalpy (− 48.1 kJ mol^−1^), might lead one to ascribe the sorption process to weak molecular interactions (i.e., physisorption), such as electrostatic attraction, ion–dipole and dispersion interactions (Lima et al. 2021). This would be certainly true for sorption from gas phase. However, the interpretation of the magnitude of Δ*H*° in liquid/solid systems is less straightforward because it reflects changes in the molecular interactions of both the solid and liquid phase arising from solvation/desolvation effects (Salvestrini et al. 2022).

The FT-IR measurements indicate alterations in the peaks of the bands corresponding to the − OH and − COOH bonds of carboxylic and phenolic groups which suggests a possible interaction of Cu(II) ions with these groups through electrostatic attraction, as also suggested by Dhaouadi et al. (Dhaouadi et al. 2021b, a) and by the fact that deprotonated –COOH groups dominate at a pH range between 2.0 and 6.0 (pH of the present work = 5.2) (Tran 2017). Such electrostatic interactions are accompained by ion exchange process. This can be inferred from the SEM/EDS compositional analysis (Fig. 3) through the reduction of the content of some exhangebale cations after Cu^2+^ uptake: for example, Ca^2+^, Mg^2+^, and above all Na^+^ ions content decrease from 9.2 to 6.12%, from 0.7% to below L.O.D. and from 7.1 to 1.2%, respectively.

The reusability tests and the high desorption degree of Cu(II) ions (≈ 80% in about 30 min) by strong acid solution treatment indicate weak bond with the sorbent, hence supporting the hypothesis of physisorption and suggesting the possibility of reusing the sorbent material several times without substantial changes in its sorption performances. A similar result was observed by Veeravelan and coworkers (Veeravelan et al. 2016) using jujube seed biochar (Zizyphus).

**Fig. 15:**
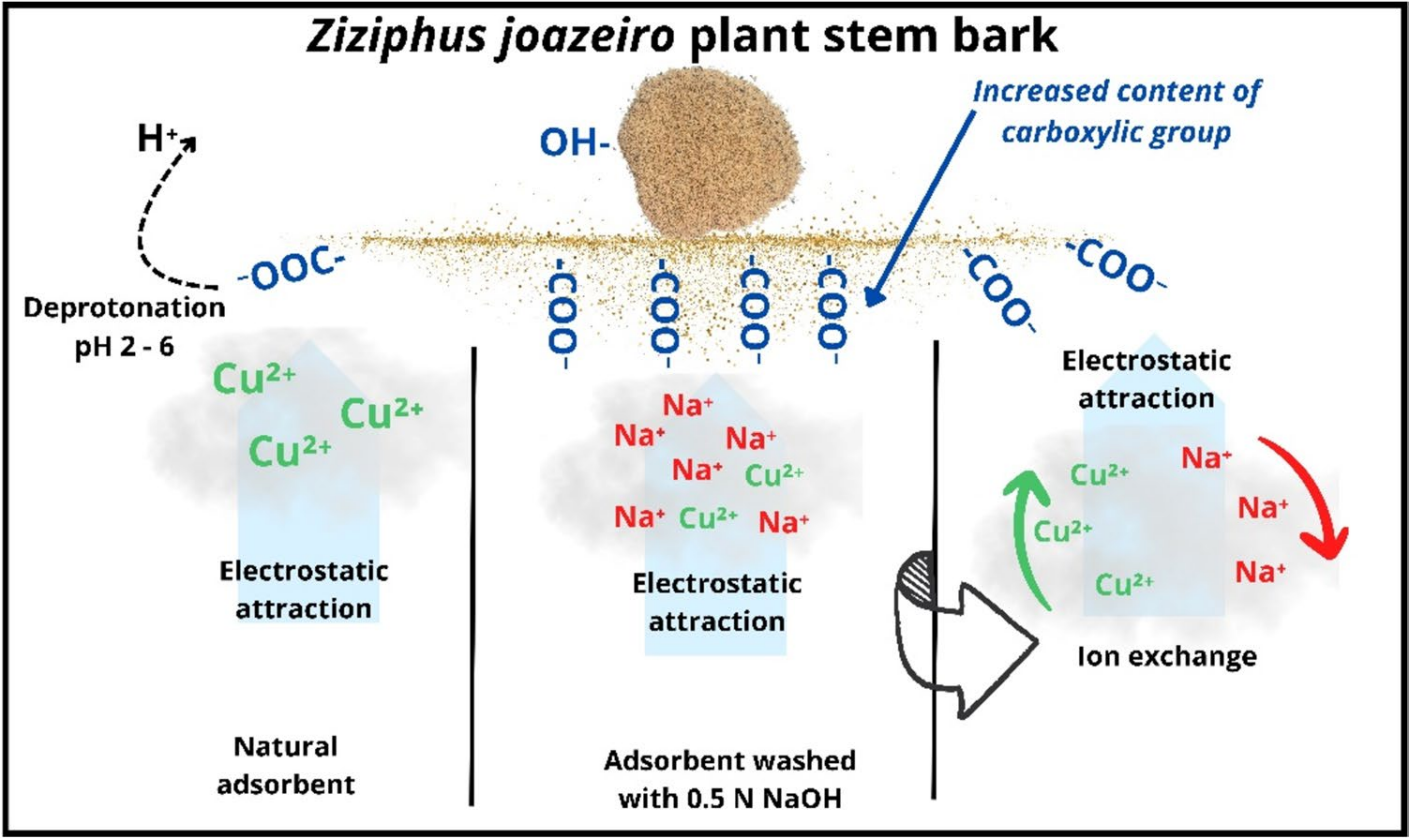
Primary mechanisms proposed for the sorption of copper onto natural and NaOH-washed *Ziziphus joazeiro* bark

The better performance of the NaOH-W sorbent in the equilibrium isotherm experiments (q_exp_ = 25.9 mg g^−1^) with respect to the material washed with water (q_exp_ = 16.5 mg g^−1^) may be related to the fact that alkaline washing promoted the enrichment of the sorbent surface with i) Na^+^ ions which are subsequently more easily exchanged, compared to the original exchangeable ions (e.g. Ca^2+^), with Cu(II) and ii) oxygenated groups such as carboxylic groups which are actively involved in copper binding (Fig. 15).

The residual undesorbed copper (≈ 20%) provides evidence of stronger interactions between Cu(II) and the sorbent which could be ascribed to complexation-chelation processes (Guo et al. 2020; Ho and Mckay 2002; Wu et al. 2021).

## Conclusion

The sorption of Cu(II) ions was investigated in batch mode using natural and modified *Ziziphus joazeiro* bark.

According to the results, sorption equilibrium was reached for most of the experiments after approximately 2 h of contact time. Intraparticle diffusion was found to be the kinetic controlling step of copper uptake.

The highest sorption capacity was achieved by washing the raw material with 0.5 N NaOH. Cu(II)/Na + ions exchange and electrostatic attractions between Cu(II) and deprotonated carboxylic and phenolic groups were identified as the primary sorption mechanisms. A secondary role in the sorption process could be played by complexation-chelation processes through carboxylic groups which likely account for the portion of copper more hardly retained by the sorbent. Competing sorption experiments revealed that sodium ion has a positive effect on the sorption of copper, whereas the opposite is true for calcium ion. The thermodynamic investigation demonstrated that the sorption process is exothermic and favorable at lower temperatures (∆H°: − 48.1 ± 13.5 kJ.mol^−1^).

The good sorption capacity of the alkaline treated bark outlines a possible route for the valorization of biomass waste derived from the extraction of bioactive compounds.

## Supplementary information

Below is the link to the electronic supplementary material.

Supplementary file1 (DOCX 3594 KB)

## Data Availability

Data will be made available on reasonable request.
