# Supplementary material for: Sorption thermodynamic and kinetic study of Cu(II) onto modified plant stem bark
Source: Environ Sci Pollut Res Int. 2024 Oct 22;31(52):61740–62. doi: 10.1007/s11356-024-35194-6 (PMC11541320; doi:10.1007/s11356-024-35194-6)
Supplement: Supplementary file 1 — Supplementary file1 (DOCX 3594 KB) [file 11356_2024_35194_MOESM1_ESM.docx]

**Sorption thermodynamic and kinetic study of Cu(II) onto modified plant stem bark**

**Supplementary Information**

Yannice Tatiane da Costa Santos^a,b^, Stefano Salvestrini^c*^, Clara Beatryz Gomes Vieira^a^, Jorge Marcell Coelho Menezes^d^, Antonio Junior Alves Ribeiro^a^, João Victor Serra Nunes^e^, Henrique Douglas Melo Coutinho^b^, Diniz Sena Júnior^b^, Francisco José de Paula Filho^d^ and Raimundo Nonato Pereira Teixeira^b^

^a)^ Federal Institute of Education, Science and Technology of Ceará – campus Juazeiro do Norte. Av. Plácido Aderaldo Castelo, 1646, 63.040-540, Juazeiro do Norte, Ceará, Brazil.

^b)^ Department of Biological Chemistry, Regional University of Cariri, R. Cel. Antonio Luis 1161, 63105000, Crato, Ceará, Brazil.

^c)^ Department of Environmental, Biological and Pharmaceutical Sciences and Technologies, University of Campania “Luigi Vanvitelli”, via Vivaldi 43, 81100, Caserta, Italy.

^d)^ Science and Technology Center, Federal University of Cariri, Av. Ten. Raimundo Rocha, 1639, 63048-080, Juazeiro do Norte, Ceará, Brazil.

^e)^Analitycal Center, Federal University of Ceará – campus Pici. Av. Humberto Monte, N/N, 60.440-900, Fortaleza, Ceará, Brazil.

*Corresponding author. Email address: stefano.salvestrini@unicampania.it

**Table S1** Error functions

| **Function** | **Equations** | **Parameters** |
| --- | --- | --- |
| R² Adjusted | $R^{2}adj.=1- \frac{\left( 1-R^{2} \right)\left( N-1 \right)}{N-P-1}$ | P: Number of model parameters  N: Sample size (number of experimental data)  R²: Coefficient of determination  q_exp_: Experimental sorption capacity (mg.g^−1^)  q_model_: Adsorption capacity calculated by model  ν: degree of freedom |
| Sum of square error | $SSE= \sum_{i=1}^{N} \left( qexp-qmodel \right)^{2}$ |  |
| Chi-square | $\chi^{2}= \sum_{i=1}^{N} \frac{\left( qexp-qmodel \right)^{2}}{qexp}$ |  |
| Akaike Information Criterion (AIC) | $AIC= 2P+N\times ln\left( \frac{SSE}{\upsilon} \right)$ |  |
| Corrected Akaike Information Criterion (AICc) | $AICc= AIC+\frac{2P^{2}+2P}{N-P-1}$ |  |

**Table S2** FT-IR Peaks and groups assignment

| Peak | Wavenumber (cm^-1^) | Assignments | References |
| --- | --- | --- | --- |
| 1 | 3396.1 e 3361.1 | -OH Stretching intra e intermolecular from cellulose, lignin and hemicellulose, and saponin. (3290 a 3416)  H-bonded and OH groups stretching from cellulose, lignin hemicellulose (3441- 3393)  Bonds H from OH and bonds de H inter e intramolecular (3800 – 3000) | (Bouchelkia et al., 2016; Guimarães et al., 2020; Kovacova et al., 2020; Lun et al., 2017; Salazar-Pinto et al., 2020) |
| 2 | 2925.8 | -CH symmetric from cellulose, lignin e hemicellulose (2923)  -CH stretching (3000 a 2800)  -CH stretching (2926)  -CH from celullose e hemicellulose (2929)  -CH stretching in methyl and methylene groups cellulose, lignin and hemicellulose (2918- 2922) | (Abreu, 2011; Al Othman et al., 2013; Javier-Astete et al., 2021; Pancholi et al., 2023; Somasekhara Reddy et al., 2012) |
| 3 | 1728.4 | C=O hemicellulose (such as esterified resin acids,waxes and fats) (1718 a 1733) | (Javier-Astete et al., 2021) |
|  |  | C=O unconjugated lignin (1720 a 1690)  C=O hemicellulose  C=O stretching vibrations in esters groups hemicellulose (1735)  C=O stretching carbonyl, carboxyl, and acetyl groups; xylans from oils, cellulose and hemicellulose (1725) | (Li et al., 2018; Lopes et al., 2018; Pancholi et al., 2023; Saliba et al., 2001) |
| 4 | 1616.1 | C=O stretching – Flavones (1621 a 1623)  C=O carbonyl group (1621)  C=O conjugated lignin (1660 a 1650)  C=O stretching vibration (1616 a 1649)  -OH beding of the absorbed water (1631 - 1647)  C=C stretching vibration and -COOH groups stretching vibration in aromatics groups associed with cellulose, lignin and hemicellulose (1610) | (Hafshejani et al., 2015; Javier-Astete et al., 2021; Li et al., 2018; Pancholi et al., 2023; Priya et al., 2022; Saliba et al., 2001) |
| 5 | 1511.9 | Aromatic skeletal vibrations (1512 a 1511)  Aromatic skeletal vibrations lignin (1500 a 1550)  Aromatic ring vibrations lignin (1606 – 1608 and 1500 – 1515)  C=C stretching vibration in aromatics rings liginin (1502 – 1516)  Aromatic skeletal vibrations (1508, 1605); C=C stretching vibrations (1504) | (Abreu, 2011; Javier-Astete et al., 2021; Li et al., 2018; Lopes et al., 2018; Pancholi et al., 2023) |
| 6 | 1369.1 | -CH bending cellulose, liginin, hemicellulose (1383 – 1385)  -CH deformation cellulose, hemicellulose (1373)  -CH_3_ symmetrical angular vibration cellulose, hemicellulose (1369) | (Li et al., 2018) |
| 7 | 1315.5 | -CH_2_ wagging cellulose (1320)  -CH_2_ bending (1323 a 1368)  -CH_2_ deformation vibration; -CH3 symmetric deformation cellulose, hemicellulose and oils (1342)  -C-C and C-O skeletal vibrations cellulose (1322 – 1332); C-O stretch of S-ring lignin (1322 – 1339) | (Javier-Astete et al., 2021; Lopes et al., 2018; Pancholi et al., 2023; Priya et al., 2022) |
| 8 | 1222.2 | C-O aromatic rings liginin (1275)  C-C, C-O and C=O stretching; C-O stretching from lignins and oils (1245)  C-O stretch of G-ring lignin (1247 – 1261) and -COO vibration of acetyl groups hemicelulose  -COO^-^ Vibration of acetyl groups hemillelulose (1236) | (Abreu, 2011; Pancholi et al., 2023; Saliba et al., 2001; Souza et al., 2020) |
| 9 | 1039.4 | C-O stretching (1048)  C-O stretching (1000 a 1200)  C-O stretching C(6) lignin (1045)  C-O stretching hollocellulose and lignin (1032 - 1021)  C-O stretching C(6) cellulose (1032)  C-O from carboxyl groups and alcohol (1027) | (Abreu, 2011; Hafshejani et al., 2015; Somasekhara Reddy et al., 2012; Souza et al., 2020) Javier-Astete et al., 2021; Krishnani & Ayyappan, 2006) |

| Natural Powder | | Natural | |
| --- | --- | --- | --- |
| 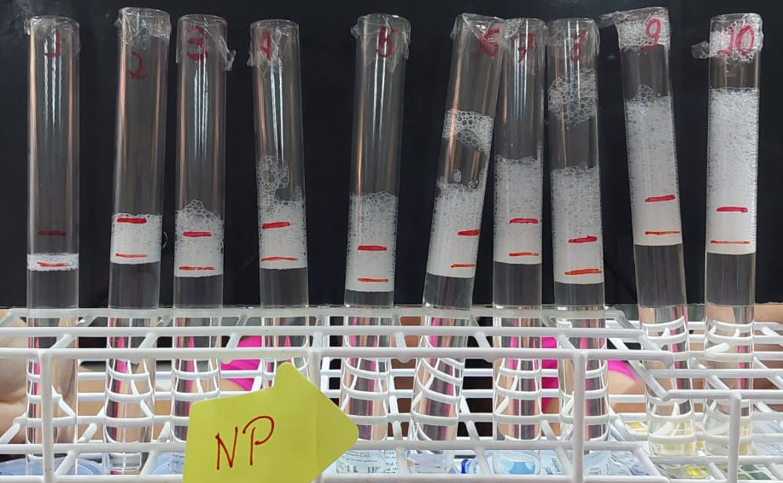 | | 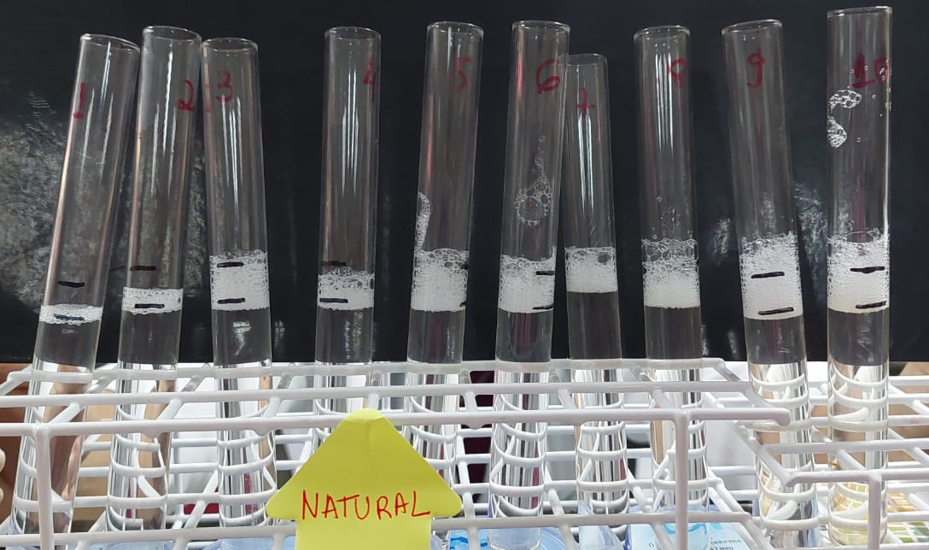 | |
| Washed by Water | Washed by NaOH | | Washed by Ethanol |
| 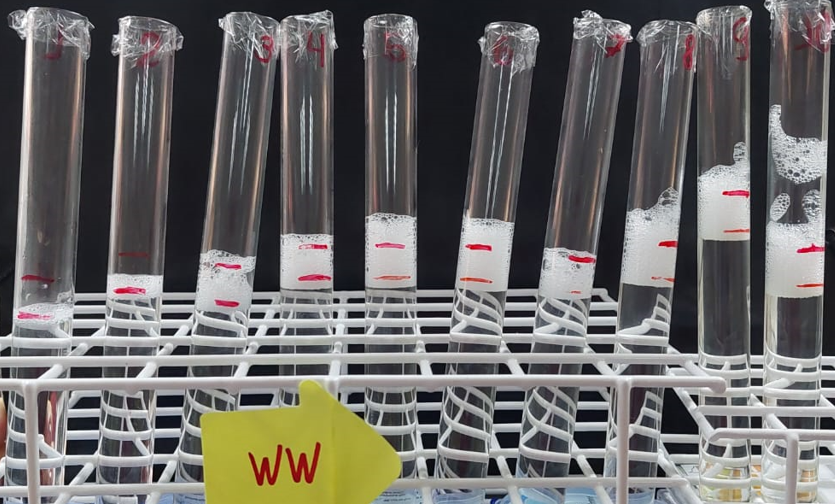 | 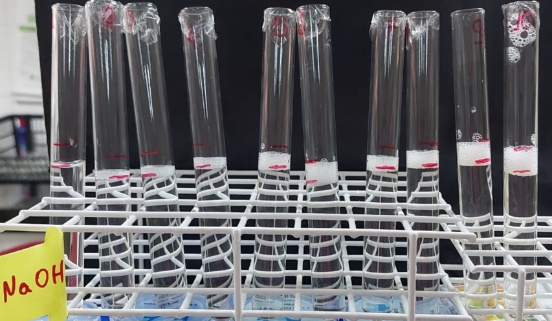 | | 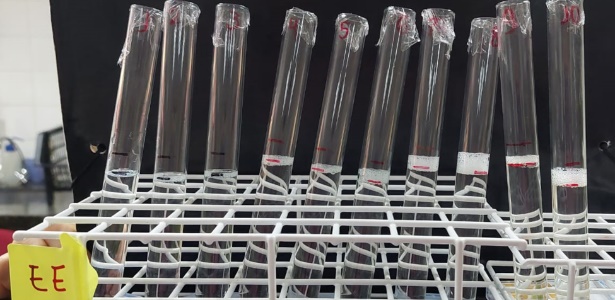 |
| Extract aspect after boiling | | | |
| 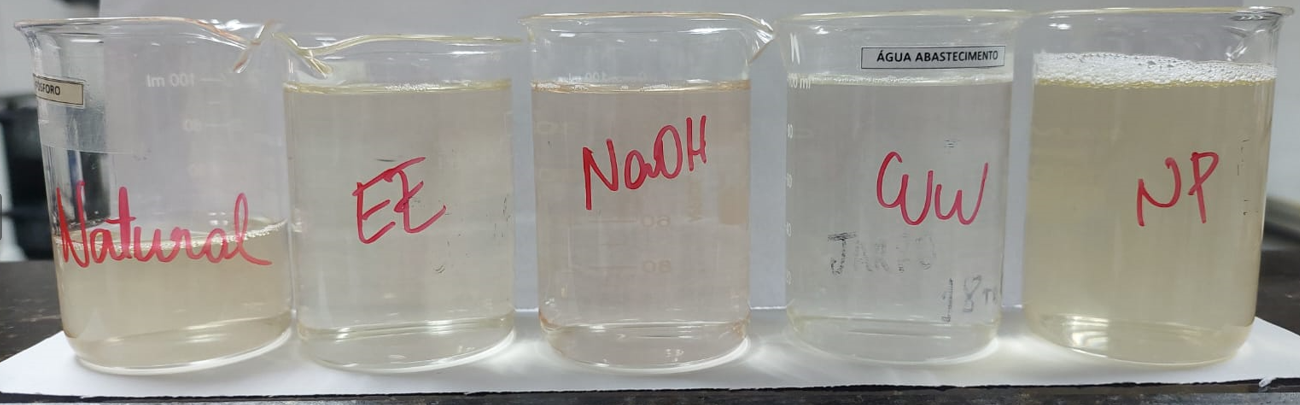 | | | |

**Figure S1** Aphrosimetric index for semi-quantification of foam (saponin).

**Table S3**

|  | Natural Powder | > Natural | > Water | > NaOH-W | > Ethanol |
| --- | --- | --- | --- | --- | --- |
| Afrosimetric Index | 998 | 665 | 499 | 369 | 200 |
| qexp (mg/g) | 4.6 | 8.4 | 7.2 | 9.9 | 6.7 |

**Figure S2** Equilibrium isotherm models applied to the adsorbent WW and NaOH, at 25°C, 100 rpm, 0.05g adsorbent and pH 5.2.

**TABLE S4** Curve fitting statistics for the isotherms modellization of NaOH and WW adsorbents at 25°C

|  | **N** | **K** | **ν** | **SSE** | AIC | AICc |
| --- | --- | --- | --- | --- | --- | --- |
| **NaOH** | | | | | | |
| **Langmuir** | 9 | 2 | 7 | 13.07 | 9.62 | 11.62 |
| **Freundlich** | 9 | 2 | 7 | 19.58 | 13.26 | 15.26 |
| **Temkin** | 9 | 2 | 7 | 13.74 | 10.07 | 12.07 |
| **Sips** | 9 | 3 | 6 | 3.65 | 1.53 | 6.33 |
| **WW** | | | | | | |
| **Langmuir** | 8 | 2 | 6 | 21.16 | 14.08 | 16.48 |
| **Freundlich** | 8 | 2 | 6 | 30.72 | 17.07 | 19.47 |
| **Temkin** | 8 | 2 | 6 | 26.50 | 15.88 | 18.28 |
| **Sips** | 8 | 3 | 5 | 21.10 | 17.52 | 23.52 |

**N:** number of the data

**K:** number model parameters

**ν:** Free degree

**SSE:** Sum of square error

**AIC and AICc**: Akaike Information Criterion, and Corrected Akaike Information Criterion

|  | **N** | **P** | **ν** | **RSS** | **AIC** | **AICc** |
| --- | --- | --- | --- | --- | --- | --- |
| **15°C** | | | | | | |
| **Langmuir** | 6 | 2 | 4 | 1.70 | -1.13 | 2.87 |
| **Freundlich** | 6 | 2 | 4 | 11.18 | 10.16 | 14.16 |
| **Temkin** | 6 | 2 | 4 | 0.83 | -5.40 | -1.40 |
| **Sips** | 6 | 3 | 3 | 0.70 | -2.75 | 9.25 |
| **25°C** | | | | | | |
| **Langmuir** | 6 | 2 | 4 | 4.62 | 4.87 | 8.87 |
| **Freundlich** | 6 | 2 | 4 | 4.42 | 4.60 | 8.60 |
| **Temkin** | 6 | 2 | 4 | 0.60 | -7.42 | -3.42 |
| **Sips** | 6 | 3 | 3 | 0.73 | -2.50 | 9.50 |
| **35°C** | | | | | | |
| **Langmuir** | 6 | 2 | 4 | 2.30 | 0.68 | 4.68 |
| **Freundlich** | 6 | 2 | 4 | 3.00 | 2.27 | 6.27 |
| **Temkin** | 6 | 2 | 4 | 0.84 | -5.39 | -1.39 |
| **Sips** | 6 | 3 | 3 | 0.59 | -3.76 | 8.24 |
| **45°C - 6 points** | | | | | | |
| **Langmuir** | 6 | 2 | 4 | 6.15 | 6.58 | 10.58 |
| **Freundlich** | 6 | 2 | 4 | 0.77 | -5.86 | -1.86 |
| **Temkin** | 6 | 2 | 4 | 3.53 | 3.25 | 7.25 |
| **Sips** | 6 | 3 | 3 | N.C.* | -5.71 | N.C.* |

**Table S5** Curve fitting statistics for the isotherms modellization of NaOH adsorbent at

various temperatures.

*No convergence.

**N:** number of the data

**K:** number model parameters

**ν:** Free degree

**SSE:** Sum of square error

**AIC and AICc**: Akaike Information Criterion, and Corrected Akaike Information Criterion

 **Figure S3** Isotherm models for the alkali-washed adsorbent at various temperatures.

References

Abreu, A. L. de. (2011). *Modificação química de resídui lignocelulósico para preparação de compósito* [Dissertação de mestrado, Universidade Federal de Larvas]. http://repositorio.ufla.br/jspui/bitstream/1/2564/1/DISSERTA%C3%87%C3%83O_Modifica%C3%A7%C3%A3o%20qu%C3%ADmica%20de%20res%C3%ADduo%20lignocelul%C3%B3sico%20para%20a%20prepara%C3%A7%C3%A3o%20de%20comp%C3%B3sito.pdf

Al Othman, Z. A., Habila, M. A., & Hashem, A. (2013). Removal of zinc(II) from aqueous solutions using modified agricultural wastes: Kinetics and equilibrium studies. *Arabian Journal of Geosciences*, *6*(11), 4245–4255. https://doi.org/10.1007/s12517-012-0672-9

Bouchelkia, N., Mouni, L., Belkhiri, L., Bouzaza, A., Bollinger, J.-C., Madani, K., & Dahmoune, F. (2016). Removal of lead(II) from water using activated carbon developed from jujube stones, a low-cost sorbent. *Separation Science and Technology*, *51*(10), 1645–1653. https://doi.org/10.1080/01496395.2016.1178289

Guimarães, M. L., da Silva, F. A. G., da Costa, M. M., & de Oliveira, H. P. (2020). Green synthesis of silver nanoparticles using Ziziphus joazeiro leaf extract for production of antibacterial agents. *Applied Nanoscience (Switzerland)*, *10*(4), 1073–1081. https://doi.org/10.1007/s13204-019-01181-4

Hafshejani, L. D., Nasab, S. B., Gholami, R. M., Moradzadeh, M., Izadpanah, Z., Hafshejani, S. B., & Bhatnagar, A. (2015). Removal of zinc and lead from aqueous solution by nanostructured cedar leaf ash as biosorbent. *Journal of Molecular Liquids*, *211*, 448–456. https://doi.org/10.1016/j.molliq.2015.07.044

Javier-Astete, R., Jimenez-Davalos, J., & Zolla, G. (2021). Determination of hemicellulose, cellulose, holocellulose and lignin content using FTIR in Calycophyllum spruceanum (Benth.) K. Schum. And Guazuma crinita Lam. *PLoS ONE*, *16*(10 October). https://doi.org/10.1371/journal.pone.0256559

Kovacova, Z., Demcak, S., Balintova, M., Pla, C., & Zinicovscaia, I. (2020). Influence of wooden sawdust treatments on Cu(II) and Zn(II) removal from water. *Materials*, *13*(16). https://doi.org/10.3390/MA13163575

Li, X., Wei, Y., Xu, J., Xu, N., & He, Y. (2018). Quantitative visualization of lignocellulose components in transverse sections of moso bamboo based on ftir macro- and micro-spectroscopy coupled with chemometrics. *Biotechnology for Biofuels*, *11*(1), 1–16. https://doi.org/10.1186/s13068-018-1251-4

Lopes, J. de O., Garcia, R. A., & de Souza, N. D. (2018). Infrared spectroscopy of the surface of thermally-modified teak juvenile wood. *Maderas: Ciencia y Tecnologia*, *20*(4), 737–746. https://doi.org/10.4067/S0718-221X2018005041901

Lun, L. W., Gunny, A. A. N., Kasim, F. H., & Arbain, D. (2017). Fourier transform infrared spectroscopy (FTIR) analysis of paddy straw pulp treated using deep eutectic solvent. *AIP Conference Proceedings*, *1835*. https://doi.org/10.1063/1.4981871

Pancholi, M. J., Khristi, A., Athira, K. M., & Bagchi, D. (2023). Comparative Analysis of Lignocellulose Agricultural Waste and Pre-treatment Conditions with FTIR and Machine Learning Modeling. *Bioenergy Research*, *16*(1), 123–137. https://doi.org/10.1007/s12155-022-10444-y

Priya, A. K., Yogeshwaran, V., Rajendran, S., Hoang, T. K. A., Soto-Moscoso, M., Ghfar, A. A., & Bathula, C. (2022). Investigation of mechanism of heavy metals (Cr6+, Pb2+, Zn2+) adsorption from aqueous medium using rice husk ash: Kinetic and thermodynamic approach. *Chemosphere*, *286*, 131796. https://doi.org/10.1016/j.chemosphere.2021.131796

Salazar-Pinto, B. M., Zea-Linares, V., Villanueva-Salas, J. A., & Gonzales-Condori, E. G. (2020). Cd (II) and Pb (II) biosorption in aqueous solutions using agricultural residues of Phaseolus vulgaris L.: Optimization, kinetics, isotherms and desorption. *Revista Mexicana de Ingeniería Química*, *20*(1), 305–322. https://doi.org/10.24275/rmiq/IA1864

Saliba, E. de O. S., Rodriguez, N. M., Morais, S. A. L. de, & Piló-Veloso, D. (2001). LIGNINAS-MÉTODOS DE OBTENÇÃO E CARACTERIZAÇÃO QUÍMICA LIGNINS-ISOLATION METHODS AND CHEMICAL CHARACTERIZATION. *Ciências Rural*, *31*(5), 917–928.

Somasekhara Reddy, M. C., Sivaramakrishna, L., & Varada Reddy, A. (2012). The use of an agricultural waste material, Jujuba seeds for the removal of anionic dye (Congo red) from aqueous medium. *Journal of Hazardous Materials*, *203–204*, 118–127. https://doi.org/10.1016/j.jhazmat.2011.11.083

Souza, L. do S. S., Pereira, A. M., Farias, M. A. dos S., Oliveira, R. L. e., Duvoisin, S., & Quaresma, J. N. N. (2020). Valorization of andiroba (Carapa guianensis aubl.) residues through optimization of alkaline pretreatment to obtain fermentable sugars. *BioResources*, *15*(1), 894–909. https://doi.org/10.15376/biores.15.1.894-909
